# Allatostatin-C signaling in the crab *Carcinus maenas* is implicated in the ecdysis program

**DOI:** 10.1242/jeb.249929

**Published:** 2025-03-17

**Authors:** Jodi L. Hoppes, David C. Wilcockson, Simon G. Webster

**Affiliations:** ^1^ School of Natural Sciences, Brambell Laboratories, Bangor University, Bangor LL57 2UW, UK; ^2^ Department of Life Sciences, Edward Llywd Building, Aberystwyth University, Aberystwyth SY23 3DA, UK

**Keywords:** Allatostatin C family neuropeptides, Receptor de-orphaning, Neuroanatomy, Release patterns during ecdysis

## Abstract

The allatostatin (AST) family of neuropeptides are widespread in arthropods. The multitude of structures and pleiotropic actions reflect the tremendous morphological, physiological and behavioral diversity of the phylum. Regarding the AST-C (with C-terminal PISCF motif) peptides, crustaceans commonly express three (AST-C, AST-CC and AST-CCC) that have likely arisen by gene duplication. However, we know little regarding their physiologically relevant actions. Here, we functionally characterize the cognate receptor for AST-C and AST-CC, determine tissue expression, and comprehensively examine the localization of AST mRNA and peptide. We also measured peptide release, circulating titers and performed bioassays to investigate possible roles. AST-C and AST-CC activate a single receptor (AST-CRd), but this, and other candidate receptors, were not activated by AST-CCC. Whole-mount *in situ* hybridization and hybridization chain reaction fluorescent *in situ* hybridization complemented neuropeptide immunolocalization strategies and revealed extensive expression of AST-Cs in the central nervous system. AST-C or AST-CCC expressing neurons were found in the cerebral ganglia, but AST-CC expression was never observed. Of note, we infer that AST-C and AST-CC are co-expressed in every neuron expressing crustacean cardioactive peptide (CCAP) and bursicon (BURS); all four peptides are released from the pericardial organs during a brief period coinciding with completion of emergence. In contrast to other studies, none of the AST-C peptides exhibited any effect on ecdysteroid synthesis or cardiac activity. However, expression of the AST-C receptor on hemocytes suggests a tantalizing glimpse of possible functions in immune modulation following ecdysis, at a time when crustaceans are vulnerable to pathogens.

## Teaser

**Highlighted Article:** The pleiotropic actions of allatostatin-C peptides are not well known. Neuroanatomical investigations, coupled with receptor de-orphaning and measurement of circulating titers, suggest previously unreported roles in the ecdysis program.

## INTRODUCTION

Neuropeptides belonging to the so-called allatostatin (AST) family, first characterized by their ability to inhibit juvenile hormone synthesis by the corpora allata in insects (Stay and Tobe, 2007), are both abundant and diverse in the panarthropods. Based on peptide sequence and conserved motifs, three classes are recognized: A, B and C (reviewed by Verlinden et al., 2015). Despite their appellations, many physiologically relevant roles, unrelated to their original biological activities, were described in insects some time ago, including (and this list is not exhaustive): for AST-A (Y/FXGL-NH_2_ motif; Pratt et al., 1989), inhibition of foregut contractility in *Leucophaea maderae* (Duve et al., 1995) and in *Diploptera punctata* hindgut (Lange et al., 1995), cardiac rhythm and inhibition of vitellogenin synthesis in *Blatella germanica* (Vilaplana et al., 1999; Martin et al., 1996), and stimulation of enzyme activity in the midgut of *D. punctata* (Fusé et al., 1999). For AST-B [W(X_6/7_) W-NH_2_ motif; Lorenz et al., 1995; Schoofs et al., 1991], also known as myoinhibitory peptide (MIP), apart from the well-known activities in inhibiting spontaneous hind-gut and oviduct contractions in *Locusta migratoria* and *L. maderae* (Schoofs et al., 1991), and hindgut contractions in *Rhodnius prolixus* (Lange et al., 2012), other activities include involvement in ecdysis behavior in *Drosophila* and *Manduca sexta* (Kim et al., 2006), inhibition of ecdysteroid synthesis by prothoracic glands of *Bombyx mori* (Hua et al., 1999), and possible involvement in the circadian clock in *Drosophila* (Kolodziejczyk and Nässel, 2011) and in *Leucophaea maderae* (Schulze et al., 2012). For AST-C (PISCF motif; Kramer et al., 1991), biological activities include inhibition of foregut contractions in *Lacanobia oleracea* (Matthews et al., 2007), stimulation of protease release in the hindgut of *Tribolium castaneum*, and modulation of nociception and immune responses in *Drosophila melanogaster* (Bachtel et al., 2018). The variety of activities of these neuropeptides clearly reflect the accepted view of neuropeptide actions in that they are pleiotropic, i.e. they have many functions unrelated to the first-described biological activities for which they were named, and that activities may be species specific, which is undoubtedly a reflection of the enormous diversity in arthropod morphology, physiology and life history strategies.

Research on the presence of these peptides in crustaceans has similarly identified a bewildering number and diversity of allatostatins in this important arthropod group (reviewed by Bendena and Tobe, 2012). This was first exemplified by an early study of AST-A peptides in the green shore crab *Carcinus maenas* (Duve et al., 1997), where 25 AST-A-type peptides were identified. With the combined use of high-resolution mass spectrometry technologies and data mining of transcriptomes, it evident that there are many more of these peptides. For example, in our model *C. maenas*, a total of 26 AST-A, 11 AST-B and 3 AST-C peptides have been described from transcriptomic analysis (Christie, 2016), eclipsing the large number of these identified by earlier analyses in this crab (Ma et al., 2009).

Although the biological activities of various AST-family peptides have been characterized in crustaceans with respect to their modulatory activities on the central patten generators of the stomatogastric (STG) and cardiac (CGN) ganglia (Dickinson et al., 2019; Szabo et al., 2011), much less is known concerning other biological activities, and this is vividly exemplified by neuropeptides in the AST-C family. Decapod crustaceans commonly express three AST-C peptides, AST-C, AST-CC and AST-CCC, the last being C-terminally amidated (Dircksen et al., 2011; Hou et al., 2015; Veenstra, 2016a), that are likely to have arisen by gene duplication (Veenstra, 2016b,c). Most insects express only AST-C and AST-CC, with the exception of *L. migratoria*, in which all three are present (Hou et al., 2015). Thus, a pertinent question to ask is, given the diversity of AST-C peptides in crustaceans, is this reflected in similar receptor diversity? Whilst two AST-C type receptors are activated by AST-C and AST-CC in *Homarus americanus* (Muscato et al., 2021), only one has been described in *Cancer borealis* and *Scylla paramamosain* (Dickinson et al., 2019; Liu et al., 2021); in these, none appear to be activated by AST-CCC.

To determine the roles of AST-C family peptides in our crustacean model *Carcinus maenas*, we took an approach that combined molecular, physiological and biochemical investigations to firstly de-orphan candidate AST-C-type receptors, ascribe relevant ligands and determine tissue expression. Second, the anatomy of neurons expressing each peptide was investigated using a combined approach of immunohistochemistry (IHC), conventional *in situ* hybridization (ISH) and hybridization chain reaction fluorescent ISH (HCR-FISH) to shed light on possible functions of these peptides and to discriminate between neuromodulatory and/or transmitter roles versus those in which neuroanatomy showed for the first time that they were released into the hemolymph as circulating neurohormones. Finally, by measuring circulating hormone levels during the molt cycle and using bioassays to investigate proposed activities of these peptides, building on the results obtained from these studies and other published work, we determined release patterns for AST-C and AST-CC during the ecdysis program to elaborate on our current model of molt control.

## MATERIALS AND METHODS

### Animals and tissue collection

Specimens of mature green shore crabs *Carcinus maenas* (Linnaeus 1758) were collected using baited traps or, if in pre-molt, by hand from the Menai Strait, UK. Crabs were maintained in a recirculating seawater system at ambient temperature and photoperiod, and fed with fish. To accurately molt stage crabs for hemolymph sampling, specimens were housed individually and staged according to Drach and Tchernigovtzeff (1967), with fine temporal staging during ecdysis according to Phlippen et al. (2000). Hemolymph samples (approximately 1–2 ml) were taken from the hypobranchial sinus using a hypodermic syringe and 19 G needle, followed by immediate snap freezing in liquid nitrogen. Nervous system tissues were dissected in ice-cold *Carcinus* saline (Saver et al., 1999), following deep anesthesia on ice (1 h), and processed for either IHC with Stefanini's fixative (Stefanini et al., 1967) (overnight at 4°C) or ISH with 4% paraformaldehyde (PFA) in phosphate-buffered saline (PBS) (overnight at 4°C), followed by dehydration in a methanol series. Nervous system samples for neuropeptide quantification and for RNA samples were dissected, frozen in liquid nitrogen and stored at −80°C. This study involved the use of invertebrates and thus was not subject to UK Home Office licensing requirements.

### Immunohistochemistry and *in situ* hybridization

Fixed nervous systems were processed for whole-mount IHC as previously described (Webster et al., 2013). Antisera raised against AST-C and AST-CCC were generous gifts from Dr P. Dickinson (Bowdoin College, Brunswick, ME, USA). These were commercially produced (Lampire Biological Laboratories. Pipersville, PA, USA). Production and specificity have been previously detailed (Christie et al., 2018). An antiserum (code SY1262) raised against synthetic AST-CC (GenScript Biotech, Piscataway, NJ, USA) was produced in rabbits (Eurogentech, Seraing, Belgium) by N-terminal conjugation to bovine thyroglobulin with 1-ethyl-3- (3-dimethylamino-propyl) carbodiimide. AST-CC (1 mg) was conjugated to agarose beads using an Aminolink^®^Plus immobilization kit (Pierce Biotechnology, Rockford IL, USA). Anti-AST-CC IgG (∼2 mg) was isolated using a Protein A–Sepharose column. The procedure for peptide immobilization and elution of affinity-purified IgG was detailed in the manufacturer's instructions and gave a yield of approximately 40% of affinity purified IgG.

Whole-mount IHC on fixed nervous systems was performed according to Webster et al. (2013). Primary antiserum dilutions were: anti-AST-C, affinity-purified AST-CC antibodies, 1:1000; and anti-AST-CCC antisera, 1:5000. For double-labeling experiments, an antiserum raised against native bursicon in guinea pigs (Eurogentech, code SYC392; as previously described by Webster et al., 2013) was used at a dilution of 1:2000. Secondary antisera were Alexa Fluor^®^ 488 goat anti-rabbit, Alexa Fluor^®^ 594 goat anti-guinea pig used at 1:750 (Life Technologies, Eugene, Oregon, USA). Preparations were mounted on cavity microscope slides with Vectashield^®^ (Vectorlabs, Newark, CA, USA), coverslipped and sealed with nail varnish. Confocal images were collected and *z*-stacked (approximately 25–30 images at 5–10 µm intervals) on a Zeiss 710 confocal microscope equipped with Zen 10 software (Carl Zeiss AG, Jena, Germany).

*In situ* hybridization was carried out using digoxygenin-labeled antisense riboprobes as previously described (Wilcockson et al., 2011). Probe synthesis was performed using primers detailed in Table S1. Preparations were mounted in 50% glycerol in PBS as described, and 3D stacks of several planes of focus were imaged using Helicon Focus 6 (HeliconSoft, Kharkiv, Ukraine). Images were cropped and resized, and adjusted for brightness and contrast using Adobe Photoshop 2023 and CorelDraw 2014.

To unambiguously determine possible differential neuronal distributions of both AST-C and AST-CC transcripts in the ventral ganglion, and AST-C, AST-CC and AST-CCC in the cerebral ganglion (CG), HCR whole-mount ISH was performed based on methodology described by Bruce et al. (2021), which was originally adapted from Choi et al. (2018). For HCR, all custom probes, hairpin amplifiers (with Alexa Fluors^®^), hybridization, amplification and wash buffers were purchased from Molecular Instruments (Los Angeles, CA, USA). Probes were designed and synthesized by the same company against *C. maenas* mRNA sequences and assigned unique identifier codes by this supplier.

Tissues were dissected under nuclease-free, ice chilled *Carcinus* saline and fixed immediately in 4% PFA in PBS for 16 h at room temperature. Following fixation, tissues were washed in PTW (PBS containing 0.1% Tween-20) for 3×10 min, dehydrated through a PTW and methanol series (33%, 66% and 100% methanol) and stored at −20°C until use. Tissues were rehydrated through the same PTW and methanol series and washed in PTW (3×10 min) before permeabilizing in detergent solution [1.0% SDS, 0.5% Tween-20, 50 mmol l^−1^ Tris-HCl (pH 7.5), 1.0 mmol l^−1^ EDTA (pH 8.0) and 150 mmol l^−1^ NaCl] for 30 min at room temperature. Tissues were subsequently prehybridized in pre-warmed hybridization buffer at 37°C for 1 h before hybridization with AST-C (lot no. RTE280, amplifier B3), AST-CC (lot no. RTE283, amplifier B1) and AST-CCC (lot no. RT662, amplifier B2) probes (1 mmol l^−1^). Probes were used at 4 µl per 100 µl hybridization buffer (40 nmol l^−1^ final probe concentration). Hybridization was performed at 37°C for 48 h. Following hybridization, probes were removed and preparations were washed in pre-warmed wash buffer at 37°C (4×15 min), followed by 3×5 min washes in 5×SSCT (5×SSC made from 20× stock; 3 mol l^−1^ NaCl and 0.3 mol l^−1^ sodium citrate, supplemented with 0.1% Tween-20). A pre-amplification step of tissues incubated in amplification buffer was carried out at room temperature for 1 h followed by hairpins in amplification buffer at room temperature for 48 h. For AST-C, hairpins B3 h1 and B3 h2 with Alexa 647 were used; AST-CC was amplified with B1 h1 and B1 h2 and Alexa 488. All hairpins (3 µmol l^−1^) were heated to 95°C for 5 min and cooled to room temperature before adding to amplification buffer at 4 µl per 125 µl (final concentration was 96 nmol l^−1^). Following amplification, preparations were washed in 5×SSCT (2×15 min followed by 2×30 min) before clearing in Vectashield Plus^®^ (Vector Laboratories, Newark, CA, USA) overnight at 4°C and mounting in the same on a depression microscope slide for confocal microscopy. Microscopy was carried out on a Leica SP8 Super Resolution laser confocal platform with inverted objectives (Leica Microsystems, Milton Keynes, UK). Dye separation was maximized by imaging in sequence for each fluor. *Z*-stack sections (2–5 µm) were analyzed using Leica LAX proprietary software before exporting as maximum projection TIFF files and processing as described earlier.

### Bioassays

To determine the possible activity of AST-C and/or AST-CC on ecdysteroid synthesis, Y-organs (YOs) were dissected from ice-anaesthetized crabs and used in the *in vitro* bioassay as detailed previously (Webster, 1986). Following 24 h incubations, culture medium was aspirated and snap frozen in liquid nitrogen prior to ecdysteroid RIA. YOs were sonicated in PBS, centrifuged (13,000 ***g*** for 5 min at room temperature) and assayed for protein concentration using a BCA protein kit (EMD Millipore, Burlington MA, USA). Protein concentrations (BSA standards) were used to normalize ecdysteroid synthesis between YO pairs.

To determine possible myotropic and/or myoinhbitory activity of AST-C, AST-CC and AST-CCC, inter-molt crabs (∼65 mm carapace width) were ice-anaesthetized and rapidly decerebrated before removing all limbs and the dorsal carapace to expose the heart and pericardial cavity. The preparation was then fitted into a bowl lined with tissue, to provide a stable saline-flooded environment. The heart was connected to a force transducer (MLT0210/A) via a micro-fishing hook (size 28) and fine nylon (0.08 mm) monofilament. Connection to a PC with Chart 4.0 software was via a Bridge Pod (ML301) and Powerlab 4/20 (AD Instruments, Castle Hill, NSW, Australia). Transducer gain was set at maximum sensitivity (200 μV). Heart preparations were initially perfused with *Carcinus* physiological saline (Saver et al., 1999) at room temperature (20°C). Once a stable output was achieved, hearts were then perfused by sequentially adding approximately 2 ml saline containing AST-C, AST-CC and AST-CCC (GenScript Biotech, Piscataway, NJ, USA), and finally adding CCAP as a positive control (all peptides were at a concentration of 10^−6^ mol l^−1^ in saline). Each peptide addition was followed by extensive washout with saline, recording heart rate and beat amplitude for approximately 2 min after application of each peptide.

### Immunoassays

#### Radioimmunoassays

For AST-C, a radioimmunoassay (RIA) was used rather than (preferable) non-radioactive immunoassays, since it proved impossible to synthesize N-terminally biotinylated peptides containing the essential Cys-Cys bridge. AST-C (300 pmol, dissolved in 10 µl 200 mmol l^−1^ phosphate buffer at pH 7.5) was radio iodinated with 9.25–12.5 MBq NaI^125^ in 10^−5^ mol l^−1^ NaOH (PerkinElmer, Boston MA), using Chloramine-T (Bolton, 1989). Following termination of the reaction (4.3 µg cysteine in 100 µl phosphate buffer) and quenching (500 µl of 0.2 mg ml^−1^ KI in phosphate buffer), radiolabeled peptide was separated from unincorporated iodide on Sep-Pak C_18_ cartridges (Waters, Milford, MA, USA), by firstly washing with 10 ml of 200 mmol l^−1^ phosphate buffer then eluting the peptide with 40% isopropanol in 500 µl fractions. Small quantities (2 µl) were then taken for counting. Specific activities of 8–16 TBq mmol^−1^ were routinely obtained.

RIA conditions were as follows: iodinated AST-C 35,000 dpm per 50 µl PBS containing 0.05% BSA and 0.02% sodium azide. An antiserum raised against *Manduca sexta* AST-C (a generous gift from Dr N. Audsley, FERA, York) was used at 1:1000 in PBS at 50 µl per tube. Standard peptides 2–2000 fmol per 50 µl and unknowns were assayed in duplicate. Tubes were first incubated for 6 h at room temperature, without tracer, which was then added, followed by 4°C incubation overnight. Bound ligand was separated from unbound using 50 µl per tube immobilized donkey anti-rabbit microcellulose suspension (Sac-Cel, Immunodiagnostic Services, Boldon, Tyne and Wear, UK), with a 30–45 min incubation at room temperature, followed by addition of 1 ml ice-cold water. Tubes were centrifuged (3300 ***g*** at 4°C 5 min) and supernatant was aspirated.

Synthetic Y^2^-CCAP (Bachem AG, Bubendorf, Switzerland) was radio-iodinated to give specific activities of approximately 26 TBq mmol^−1^ as detailed previously (Phlippen et al., 2000), and used in a RIA using antiserum R3TB (provided by Prof. H. Dircksen, Zoomorphologie, Stockholm University). CCAP standards were in the range 200–1.56 fmol tube^−1^. All tubes were assayed in duplicate.

Hemolymph samples were gently thawed, centrifuged (3300 ***g*** at 4°C for 5 min), diluted with an equal volume of water and purified on Strata-X columns (33 µm polymeric reverse phase 200 mg) by elution on a vacuum manifold (Phenomenex, Macclesfield, UK). After washing with 5 ml water, peptides were eluted with 3 ml 40% isopropanol and dried by vacuum centrifugation. Recoveries were >90%.

Ecdysteroid RIAs were performed on 20 µl aliquots of culture medium using the following conditions: 100 μl 1:2000 anti-ecdysteroid serum HB-2E (a kind gift from R. D Watson, Alabama University), 100 µl H^−3^ Ponasterone A [24, 25, 26, 27-3H (N), 3.5 TBq mmol^−1^ (American Radiochemical Company, St Louis, MO, USA)] at 28,000 dpm per tube. 25-Deoxyecdysone (a kind gift from Prof. R. Lafont, Sorbonne University, Paris) was used as a standard (range 2500–19.5 pg per tube), and unknowns and standards were assayed in triplicate. Bound was separated from free using 50 µl per tube immobilized donkey anti-rabbit nitrocellulose suspension (Sac-Cel), as detailed for the AST-C RIA. Pellets were resuspended in 600 µl Hi-Safe 3 (Perkin Elmer) prior to counting. Using this RIA, both ecdysone and 25-deoxyecdysone (the two ecdysteroids synthesized by *C. maenas* YOs) were equally recognized.

#### Enzyme immunoassays

Direct (non-competitive) EIAs were used to determine specificity and cross-reactivity of the antisera (AST-C and AST-CC). Both peptides were reconstituted in 0.1 mol l^−1^ bicarbonate buffer and coated on 96-well high binding microplates (Costar 3590 Corning, VWR International, East Grinstead, UK). Standards were AST-C and AST-CC 100–0.2 pmol per 100 µl, assayed in duplicate. After overnight incubation at 4°C, plates were washed 3× in bicarbonate buffer, blocked with 0.1% BSA in 50 mmol l^−1^ TRIS at pH 8.0 for 1 h at room temperature. AST-C and AST-CC antisera were used at 1:1000 and 1:8000, respectively. After incubation overnight at 4°C (diluted in PBST, 100 µl per well), plates were washed five times in TR-FIA buffer (PerkinElmer, Waltham, MA, USA), incubated overnight at 4°C in 1:5000 goat-anti rabbit peroxidase at 100 µl per well (Vector Labs, Burlingame CA, USA), washed six times in TR-FIA buffer and developed in 1-step Ultra TMB-ELISA^®^ reagent (Thermo Scientific, Rockford, IL, USA). Color development was stopped by addition of 100 µl per well of 0.16 mol l^−1^ sulfuric acid; absorbances were measured at 450 nm.

#### Time-resolved fluoroimmunoassays

TR-FIA for bursicon was performed as detailed by Webster et al. (2013).

### Peptide purification and HPLC

To determine elution profiles of immunoreactive fractions corresponding to AST-C and AST-CC, purified pooled hemolymph samples (6.5 ml) taken from crabs at the moment of complete exuviation (E100) were reconstituted in 200 µl of 2 mol l^−1^ acetic acid, centrifuged (14,000 ***g*** for 5 min at room temperature) and immediately injected into a high-pressure liquid chromatography (HPLC) (Dionex Summit, Dionex, Sunnyvale, CA, USA). The HPLC conditions were as follows: 4.6×300 mm Jupiter C_18_ 300 Å column (Phenomenex, Macclesfield, UK), 40–80% solvent B over 40 min at 1 ml min^−1^ and detection at 210 nm (solvent A is 0.11% trifluoroacetic acid (TFA); solvent B is 60% acetonitrile and 0.1% TFA). Fractions (1 ml) were immediately dried by vacuum centrifugation, followed by reconstitution in PBS, sonication and RIA in duplicates (0.8 ml hemolymph equivalents per tube). Elution profiles of synthetic peptides were determined by separation of 10 pmol of peptide, followed by RIA, for each fraction. To determine cross-reactivity of antisera to AST-C and AST-CC, pericardial organs (POs) were rapidly dissected, extracted with 2 mol l^−1^ acetic acid and homogenization by sonication, centrifugation and HPLC separation (as above). Fractions were reconstituted in 0.1 mol l^−1^ bicarbonate buffer to give concentrations of 2 PO equivalents per well (100 µl) in the direct EIA.

### Isolation and cloning cDNA encoding AST-CR: receptor assays, RNA extraction and cDNA synthesis

Total RNA was extracted from frozen tissues using TRIzol (Invitrogen, Carlsbad, CA, USA) according to the manufacturer's instructions, followed by removal of gDNA with DNase 1 (TURBO DNA-free kit, Invitrogen). mRNA was purified using Dynabeads^®^ Oligo (dT)25 (Dynal, Oslo Norway) and stored in 10 mmol l^−1^ Tris-HCl at −80°C. First-strand cDNA synthesis was carried out using Tetro cDNA synthesis kit (Bioline, UK), using a mix of random hexamers and oligo (dT) primers under the following conditions: 25°C for 10 min, 45°C for 30 min and 85°C for 5 min.

### PCR

Endpoint PCR to determine tissue distribution of AST-CR transcripts was performed on cDNA prepared from neural tissues (eyestalk, cerebral ganglia, ventral ganglia, stomatogastric ganglia and cardiac ganglia) and non-neural tissues [YO, heart, midgut gland, gill, sperm–vas deferens, mature (stage 4) ovary, dactyl muscle, hindgut, epidermis and hemocytes]. PCR was performed in 25 µl volumes using Dream Taq^®^ DNA polymerase (Thermo Fisher Scientific, Vilnius, Lithuania) as follows: 98°C for 5 min, followed by 40 cycles of 95°C for 30 s, 58°C for 30 s, and 72°C for 45 s, with a final extension of 72°C for 5 min. Products were electrophoresed on 2% agarose gels. cDNA quality was assessed by PCR (30 cycles) using primers for arginine kinase. Primer sequences are shown in Table S1.

### Receptor cloning

The transcriptome of neural tissue of *C. maenas*, which has been previously assembled (Oliphant et al., 2018), identified four putative AST-C receptor candidates: TR71151a-d (Oliphant et al., 2018). These were chemically synthesized with a modified 5′ Kozak sequence (GAATTCGCCACC) and cloned in frame into a pcDNA3.1(+) plasmid vector (Genscript, Piscataway, NJ, USA). Separate aliquots of OneShot TOP10^®^ *E. coli* cells (Invitrogen, Paisley, UK) were transformed with the plasmids and grown on LB plates containing 100 µg ml^−1^ ampicillin overnight at 37°C. Positive clones were cultured overnight in LB broth with 100 µg ml^−1^ ampicillin at 37°C and plasmids extracted using a Qiagen Plasmid Midiprep kit according to the manufacturer's instructions. Purified plasmids were re-sequenced (MWG Eurofins, Ebersberg, Germany) and analyzed using Geneious 9.1.8 (Kearse et al., 2012).

### Cell culture and receptor assays

Chinese hamster ovary (CHO-K1) cells containing stably expressed apoaequorin (Perkin Elmer, Boston, MA) and either Gα16 or G*q* subunit (control cells) were cultured in Dulbecco's Modified Eagle Medium (DMEM) F-12 Nutrient Mixture Glutamax (Gibco^®^) supplemented with 10% fetal bovine serum (Gibco^®^). Cells were maintained in vented T75 flasks at 37°C in 5% CO_2_. Cells grown in a monolayer to approximately 60% confluency were transfected with pcDNA 3.1 constructs using FugeneHD^®^ (Promega) according to the manufacturer's recommendations. Transfection medium was prepared by combining 800 µl Opti-MEM^®^ (Gibco) with 10 µg vector and the cells further incubated overnight. Cells were detached from the culture flask by incubating for 10 min in 5 ml 0.2% EDTA in PBS, washed in 10 ml clear DMEM–F-12 containing L-glutamine and 15 mmol l^−1^ HEPES, centrifuged (for 5 min at 260 ***g***) and resuspended at a concentration of 5×10^6^ cells ml^−1^ in 0.2 µm filtered BSA medium (DMEM–F-12 containing L-glutamine, 15 mmol l^−1^ HEPES and 0.1% BSA). Coelenterazine *h* (Invitrogen, Paisley, UK) was added to give a concentration of 5 µmol l^−1^, and the cells incubated in the dark at room temperature with gentle rocking. Before the assay, cells were further diluted (10-fold) and incubated for 60 min before use. Synthetic peptides used in the assay are listed in Table S2. Dried aliquots of peptides were reconstituted in BSA medium, and 50 µl added in quadruplicate to a white 96-well plate (Optiplate^®^, Perkin Elmer). Cell suspensions were gently stirred and injected (50 µl per well) using a Mithras LB 940 microplate reader (Berthold Technologies, Bad Wildbad, Germany) and light emission (Ca^2+^ response recorded for 30 s). Cells were then lysed by injection of 50 µl per well 0.3% Triton-X in BSA medium, and light emission recorded for a further 10 s to measure total Ca^2+^ response. BSA medium was used for blank measurements (six replicates per plate) and transfection with empty vectors was used for negative controls. Data were analyzed using Mikrowin 2010 v5 (Mikrotek Laborsysteme, Overath, Germany) and SigmaPlot v15 (Systat Software, Grafiti, Slough, UK).

## RESULTS

### Identification of and functional de-orphaning the AST-C receptor

**Fig. 1.**
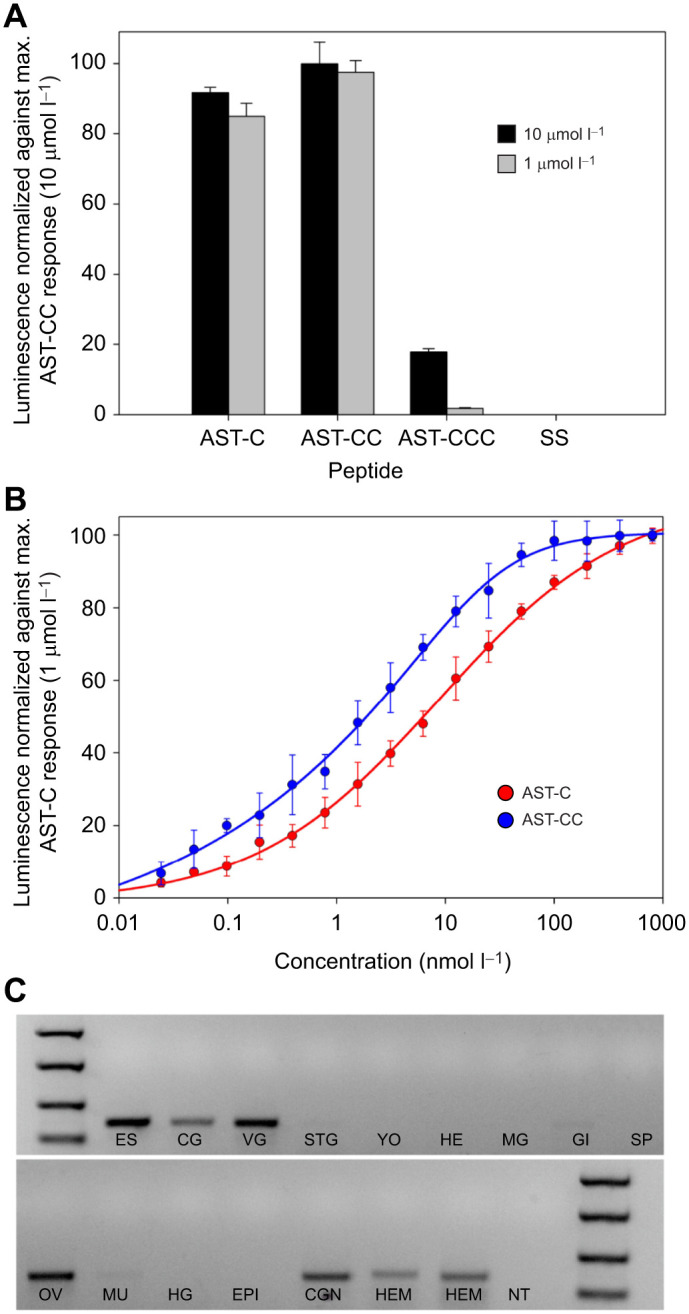
**AST-C receptor deorphaning, tissue distribution of transcripts for AST-C and AST-C receptor.** (A) Luminescence responses of CHO-K1-Aeq-Gα cells transiently expressing AST-CRd (TR71151; Oliphant et al., 2018) to *C. maenas* AST-C group peptides and the vertebrate paralog somatostatin (SS) at saturating doses (10 µmol l^−1^, black bars; 1 µmol l^−1^ grey bars). Assays were performed in quadruplicate. Data are mean+s.d. Luminescence values were normalized to the maximum luminescent response obtained with 10 µmol l^−1^ AST-CC. (B) Dose–response curves showing luminescence responses following the addition of *C. maenas* AST-C (red) or AST-CC (blue) to CHO-K1-Aeq-Gα 16 cells transiently expressing AST-CRd. Samples were assayed in quadruplicate. Data are means+s.d. from two independent experiments. Approximate ED_50_ values: AST-C, 6 nmol l^−1^; AST-CC, 2 nmol l^−1^. (C) End-point PCR showing expression of AST-CRd in tissues. mRNA isolation, cDNA synthesis and PCR conditions are as detailed in the text. ES, eyestalk; CG, cerebral ganglion; VG, ventral ganglion; STG, stomatogastric ganglion; YO, Y organ; HE, heart; MG, midgut gland; GI, gill; SP, sperm duct and/or spermatophores; OV, ovary; MU, muscle; HG, hindgut; EPI, epidermis; CGN, cardiac ganglion; HEM, hemocytes; NT, no template control. Markers are 2000, 1000, 500 and 250 bp.

Four putative AST-C peptide family receptors were identified from our *C. maenas* CNS transcriptomes (Oliphant et al., 2018). These were all cloned and each transiently expressed in CHO-K1-Aeq cells, but only AST-CR(d) was activated by AST-C peptides. Saturating doses (10, 1 µmol l^−1^) of both AST-C and AST-CC were equally active, but AST-CCC (10 µmol l^−1^) showed little activity (less than 20% of the maximum response elicited by 10 µmol l^−1^ AST-CC), and the vertebrate homolog, somatostatin, failed to activate this receptor (Fig. 1A). Dose–response relationships for AST-C and AST-CC showed that both peptides were similarly active, although the ED_50_ for AST-CC (2 nmol l^−1^) was slightly, but consistently, lower than that of AST-C (6 nmol l^−1^) (Fig. 1B). Transfection of the AST-CR(d) construct into control cells (expressing a Gq subunit) and exposure to AST-C and AST-CC produced no luminescent response.

Tissue distribution of AST-CR transcripts was determined by end-point PCR. As expected, these were expressed at extremely low levels: 40 cycles of PCR were needed to visualize products. A negative and contrast-enhanced image is shown in Fig. 1C. All tissues in the CNS gave unambiguous PCR products, as did ovarian tissue (stage 4 mature oocytes), cardiac ganglion and hemocytes. Very faint bands were observed for STG, gill and muscle samples, but the possibility that these represented trace gDNA cannot be excluded.

**Fig. 2.**
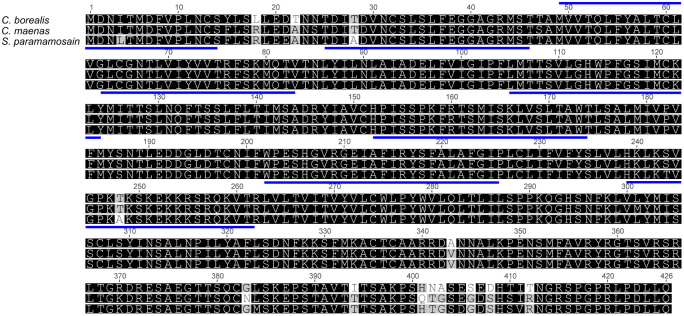
**Amino acid sequences of crab allatostatin-C receptors.** Predicted transmembrane regions (Deep TMHMM) are indicated by blue bars above the sequences. *Cancer borealis*, QCB19934.1 (Dickinson et al., 2019); *Scylla paramamosain*, MK314113 (Liu et al., 2019).

**Fig. 3.**
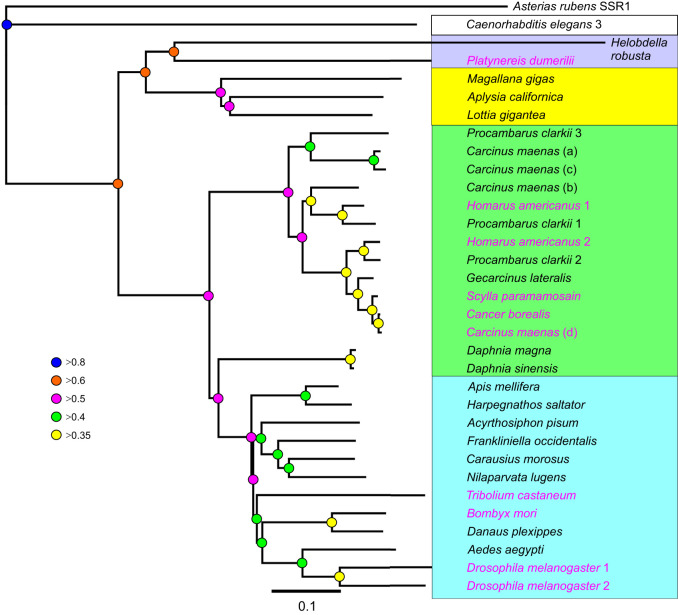
**Phylogram of AST-C receptor homologs.** Phylogram of putative and functionally de-orphaned (magenta) AST-CR homologs of selected crustaceans (green box), insects (blue box), mollusks (yellow box), annelids (lilac box) and a nematode (white box). Outgroup: Echinodermata, *Asterias rubens* somatostatin receptor 1. Sequences were trimmed to include only the predicted seven-transmembrane and loop domains. Phylograms were assembled using Geneious V.8 tree builder, using a Jukes–Cantor model with the neighbor-joining default setting. Bootstrap values at each node are shown (inset). Accession numbers are as follows: *Asterias rubens* somatostatin receptor 1, QIM61770.1 (Zhang et al., 2020); *Caenorhabditis elegans*, NP_510833.3; *Helobdella robusta*, XP_0090200674; *Platynereis dumerilii*, AKQ62999.1 (Bauknecht and Jékely, 2015); *Magallana gigas*, XP_0011429560; *Aplysia californica*, XP_005095139; *Lottia gigantea*, XP_009065270.1; *Carcinus maenas* a–d, TR71151a–d (Oliphant et al., 2018); *Procambarus clarkii* 1-3, UVI03433.1, UVI03434.1 and UVI03435.1; *Homarus americanus* 1 and 2, transcripts 10681 and 10683 (Christie et al., 2015; Muscato et al., 2021); *Gecarcinus lateralis*, transcript A5b (Tran et al., 2019); *Scylla paramamosain*, transcript A5 (Bao et al., 2018); *Cancer borealis*, QCB19934.1; *Daphnia magna*, XP_032786073.1; *Daphnia sinensis*, KAI9560913; *Apis mellifera*, XP_006560939.1; *Harpegnathos saltator*, EFN80627.1 (Bonasio et al., 2010); *Acyrthosiphon pisum*, XP_001950448.1; *Frankliniella occidentalis*, KAE8749776.1; *Carausius morosus*, AOV81581.1; *Nilaparvata lugens*, BAO01050.1; *Tribolium castaneum*, NP_001280521.1 (Audsley et al., 2013); *Bombyx mori*, NP_001127736.1 (Yamanaka et al., 2008); *Danaus Plexippus*, XP_032513530; *Aedes aegypti*, QBC65462.1; *Drosophila melanogaster* 1 and 2, NP_649040.2 and NP_001303398.1.

Amino acid sequence comparisons for the three functionally identified crab AST-CRs in *C. maenas*, *C. borealis* and *S*. *paramamosain*, shows that they are almost completely identical (Fig. 2). A phylogram constructed using AST-R sequences from insects and crustaceans is shown in Fig. 3.

### Immunohistochemistry and *in situ* hybridization

**Fig. 4.**
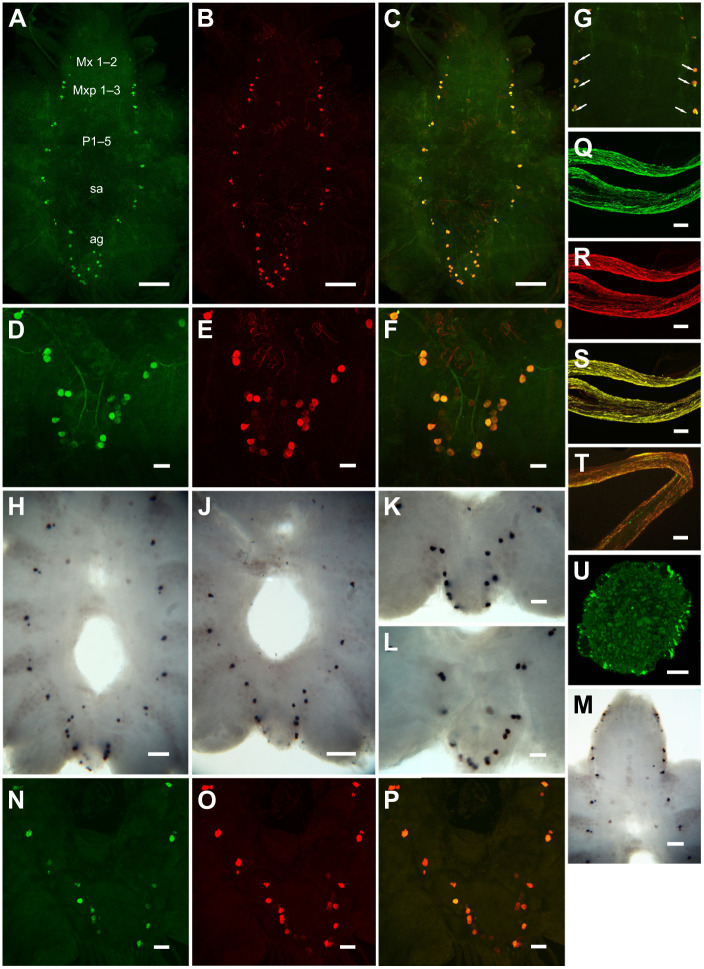
**Distribution of AST-Cs in the central nervous system of *Carcinus maenas*.** (A–P) Proteins were visualized using confocal immunohistochemistry (IHC) (A–G,Q–U); mRNA were visualized using *in situ* hybridization (ISH) (H–P). (A) Tiled whole-mount image of a ventral ganglion showing segmentally iterated pairs of AST-C and AST-CC perikarya in the sub-esophageal ganglion and thoracic ganglion, and unpaired but segmentally iterated perikarya in the abdominal ganglion. (B) Preparation as in A, labeled using BURS antiserum. (C) Merged image of A and B. (D–F) Double-immunolabeled wholemounts of abdominal ganglia showing AST-C and AST-CC immunoreactive neurons (D), BURS (E) and a merged image (F). (G) Double-immunolabeled wholemount (for AST-C and AST-CC, and BURS) of a sub-esophageal ganglion showing pairs of segmentally iterated neurons. For cells in the maxilliped neuromeres, the small cells showed little BURS immunolabeling (arrows). (H,J). Whole-mount ISH preparations of ventral ganglia hybridized with antisense probe for AST-C (H) or AST-CC (J). (K,L) Whole-mount ISH preparations of abdominal ganglia hybridized with antisense probe for AST-C (K) or AST-CC (L). (M) Whole-mount ISH preparation of a sub-esophageal ganglion hybridized with antisense probe for AST-C. (N–P) Whole-mount ISH of ventral ganglia, hybridized with HCR probes for AST-C (N) and AST-CC (O), and a merged image (P). (Q–S) Whole-mount preparations of dorsal and ventral pericardial organ trunk nerves immunolabeled for AST-C and AST-CC (Q), BURS (R) and a merged image (S). (T) Pericardial organ trunk dual immunolabeled with affinity-purified anti-AST-CC and BURS, shown as a merged image. (U) Semi-thin section (1 µm) of pericardial organ trunk, immunolabeled for AST-C and AST-CC showing axon profiles and secretory boutons on the surface of the trunk. Mx1 and 2, maxilliped neuromeres 1 and 2; Mxp 1–3, maxilliped neuromeres 1–3; P1–5, pereiopod neuromeres 1–5; ag, abdominal ganglion; sa, sternal artery. Scale bars: 500 µm in A–C; 100 µm in D–U.

Whole-mount confocal IHC and ISH of the ventral ganglion and POs is shown in Fig. 4. AST-C and AST-CC immunopositive cell bodies were observed in every neuromere of the ventral ganglion (Fig. 4A). Every neuron also expressed BURS (Fig. 4B). Merged images are shown in Fig. 4C. Colocalization of AST-C and AST-CC with BURS is shown in detail for the abdominal ganglia neurons (Fig. 4D–F) and suboesophageal ganglion (Fig. 4G). Although both AST-C and AST-CC, and BURS were similarly expressed in both the large type-1 (cdc) and small type-2 (cdn) neurons (nomenclature according to Dircksen, 1998), some differential expression was consistently seen in the sub-esophageal ganglion for the neurons in the maxilliped neuromeres (Fig. 4G). Conventional (DIG-labeled riboprobes) whole-mount ISH showed that mRNA for both AST-C and AST-CC were likely co-expressed in the same neurons (Fig. 4H–L), and this was unambiguously confirmed using HCR ISH: hybridization using antisense probes for AST-C (Fig. 4N) and AST-CC (Fig. 4O), and a merged image (Fig. 4P) show complete co-expression in all abdominal ganglion neurons. Whole-mount IHC of the POs showed complete colocalization of AST-C and AST-CC with BURS (Fig. 4Q–S), and using the affinity purified AST-CC antiserum demonstrated that immunopositive structures in the POs also colocalized with BURS (Fig. 4T). Localization of secretory boutons containing AST-C and AST-CC on the surface of the POs was confirmed on semi-thin resin-embedded sections (Fig. 4U).

**Fig. 5.**
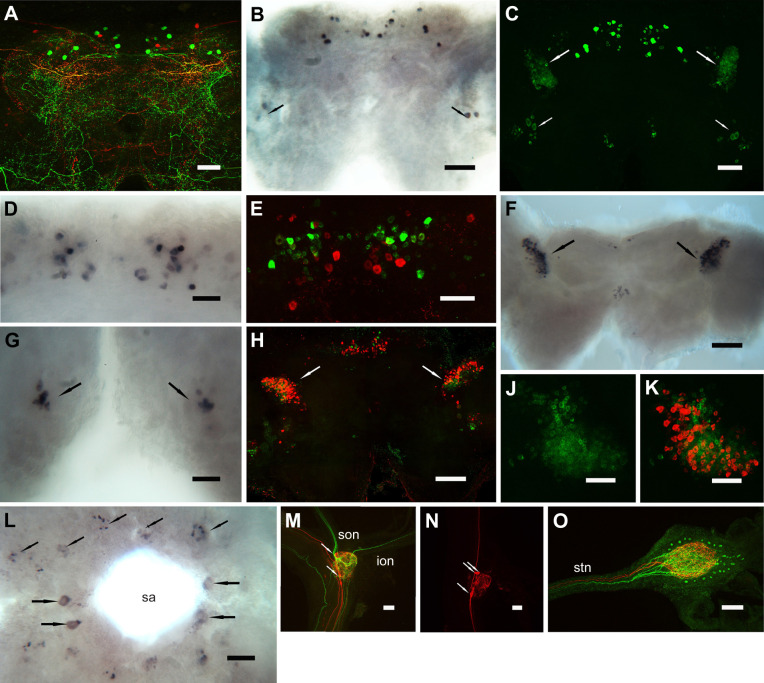
**Distribution of AST-Cs in the central nervous system of *C. maenas*.** (A–O) Proteins were visualized using confocal immunohistochemistry (IHC) (A,M–O); mRNA were visualized using *in situ* hybridization (ISH) (B–L). (A) Tiled whole-mount of the cerebral ganglion showing the dorsal aspect, dual immunolabelled for AST-C and AST-CC (green), and AST-CCC (red). There is complete separation of immunopositive structures and complex arborizations. (B) Whole-mount ISH preparation of a cerebral ganglion hybridized with antisense probe for AST-C. (C) Whole-mount HCR ISH preparation of a cerebral ganglion hybridized with an antisense probe for AST-C. Large white arrows indicate many small (10–15 µm) cell bodies of the dorsal lateral cell group; small arrows indicate small numbers of cell bodies in the posterior lateral cell group. Some of these (two pairs, ∼20–30 µm) were observed by conventional ISH shown in B (small arrows). (D) Whole-mount ISH using antisense AST-CCC probe of a dorsal view of the cerebral ganglion anterior medial cell group showing numerous cells with both strongly or weakly hybridization signals. (E) A whole-mount dual-label HCR ISH preparation of a cerebral ganglion hybridized with antisense probes for AST-C (green) and AST-CCC (red). (F) A whole-mount ISH preparation (dorsal view) of a cerebral ganglion hybridized with an antisense probe for AST-CCC. Strong hybridization signals were observed in the dorsolateral cell group (arrows). In this preparation, few hybridizing cells in the anterior medial cell group are seen. Small arrows indicate a group of hybridizing cells in the posterior medial cell group. (G) Ventral view of the tritocerebrum of a whole-mount cerebral ganglion preparation hybridized with an AST-CCC antisense probe. Two groups of small neurons (∼10–15 µm, arrows) were seen. (H) A whole-mount dual-label HCR ISH preparation of a cerebral ganglion hybridized with antisense probes for AST-C (green) and AST-CCC (red). (J,K) Arrows in H indicate groups of cells in the dorsal lateral cell group that gave weak hybridization signals for AST-C mRNA (J), but much stronger hybridization signals for AST-CCC mRNA (K). (L) Whole-mount ISH for AST-CCC of a ventral ganglion, dorsal view, anterior to the right. Four large (40–50 µm) weakly hybridizing neurons (four larger arrows) are visible and five pairs of groups of serially iterated neurons corresponding to thoracicomeres 4–8 (five smaller arrows). (M) Dual-immunolabeled wholemount of a commissural ganglion showing AST C and AST-CC immunoreactive axons and arborizations (green), and AST-CCC axons, arborizations and two large (20–30 µm) perikarya (red) (arrows). (N) A commissural ganglion immunolabeled for AST-CCC. In this preparation, three intensely labeled perikarya (arrows) are observed. (O) A whole-mount double-labeled stomatogastric ganglion showing axons in the stomatogastric nerve (stn) and dendrites, together with AST-C and AST-CC immunoreactive axons (approximately six) and dendrites, and AST-CCC immunoreactive axons (two) and dendrites. Twenty-eight small (∼10 µm) glial? cells were labeled for AST-C and AST-CC. ion, inferior esophageal nerve; son, superior esophageal nerve; sa, sternal artery. Scale bars: 100 µm in A–L,O; 50 µm in M,N.

Whole-mount confocal IHC and ISH of the cerebral, thoracic, connective and STG is shown in Fig. 5. Double immunolabeling for AST-C and AST-CC and AST-CCC in the CG revealed complex arborizations and cell bodies that showed no overlapping expression (Fig. 5A). Conventional ISH showed remarkably similar expression patterns to the AST-C and AST-CC-expressing neurons in the CG, except that two pairs of neurons were invariably seen in the posterior of the CG. Using the much more sensitive HCR-FISH confirmed that these neurons expressed only AST-C mRNA. In addition, there were several immunopositive cell bodies in the posterior lateral cell group and, in particular, low level expression of AST-C in large numbers of small diameter (10–15 µm) neurons corresponding to the dorsal lateral cell group (nomenclature according to Sandeman et al., 1992). AST-CCC ISH revealed numerous cells in the anterior medial cell group. The non-overlapping expression patterns for both AST-C and AST-CCC mRNA is shown by HCR-FISH (Fig. 5E). Conventional ISH for AST-CCC showed intense hybridization signals in the dorsolateral cell group, with hundreds of small (10–15 µm) cells, reminiscent in distribution to those expressing AST-C. However, double labeling HCR-FISH showed that these distributions did not overlap (Fig. 5H,J,K). Whilst a group of cells corresponding to the posterior medial cells (Fig. 5F) was seen in conventional ISH, these cells were not observed by HCR-FISH. On the ventral aspect of the CG tritocerebrum, two groups of small (approximately 20 µm) cells were seen, but these were not further investigated by HCR. In the ventral ganglion (VG), many very faintly hybridizing cells surrounding the sternal artery foramen were seen by conventional ISH (Fig. 5L), comprising four large (30 µm) and several groups of small (10–15 µm) cells corresponding to thoracicomeres 4–8. However, these could not be convincingly confirmed by HCR.

Whole-mount IHC of the commissural ganglia (COG) and STG double labeled for AST-C, AST-CC and AST-CCC are shown (Fig. 5M–O). Prominent descending axons containing both peptides were seen in the connectives; in the COG, a complex pattern of dendrites with overlapping peptide distributions was seen (Fig. 5M,N). For AST-CCC, at least three large (approximately 50 µm) cell bodies were seen (Fig. 5N). AST-C and AST-CC axons were observed in both the superior and inferior esophageal nerve (Fig. 5M), but AST-CCC-containing axons were not seen. However, these were obvious in double-labeled STG preparations, where six AST-C and AST-CC and two AST-CCC axons were seen in the stomatogastric nerve (Fig. 5O). In the STG, many branching arborizations were seen; notably, for AST-C and AST-CC, 28 small diameter neurons (approximately 10 µm) were seen. Unfortunately, ISH to confirm the identity of these cells was unsuccessful.

### Release patterns of AST-C during ecdysis

**Fig. 6.**
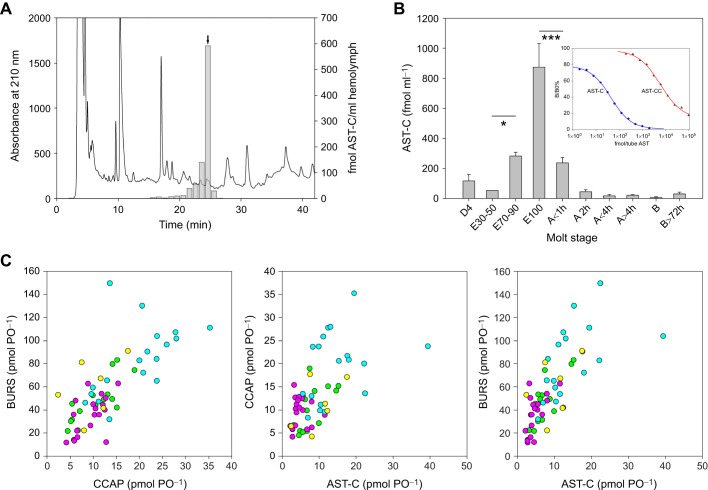
**AST-C levels in the hemolymph, and comparison with amounts of CCAP and BURS in the pericardial organs.** (A) High-pressure liquid chromatography–radioimmunoassay (HPLC–RIA) of AST-C immunoreactive fractions from 6.5 ml Strata-X purified E100 hemolymph. 0.8 ml equivalents were assayed in duplicate. Chromatographic conditions: solvent A, 0.11% trifluoroacetic acid (TFA); solvent B, 60% acetonitrile containing 0.1% TFA. Phenomenex Jupiter 300×4.8 mm C18 column, 40–80% solvent B over 40 min, 1 ml min^−1^. Retention time of HPLC–RIA of 10 pmol AST-C is shown (arrow). (B) Levels of AST-C in hemolymph over ecdysis and post-molt. Data are means±s.e.m. *N* values are as follows: D_4_, 7; E_30–50_, 5; E_70–90_, 6; E_100_, 15; A<1 h, 9; A 2 h, 6; A<4 h, 5; A>4 h, 6; B>72 h, 5. *P<0.05 (Welch's *t*-test); ***P<0.001 (Mann–Whitney rank sum test). Inset shows typical standard curves for AST-C (blue) and AST-CC (red). Cross-reactivity of AST-CC 0.25% at 50% binding. ED_50_ AST-C, 20 fmol per tube; AST-CC ED_50_, 8000 fmol per tube. (C) Scattergrams of pericardial organ (PO) peptide contents throughout the molt cycle. AST-C, BURS and CCAP were measured from extracts of paired POs and compared pairwise. A–B (green), *n*=15; C1–3 (pink), *n*=19; D2–3 (cyan), *n*=18; Ecdysis (yellow), *n*=6.

A RIA was developed to measure release patterns of AST-C during ecdysis. Since the existing antiserum to crustacean AST-C was unsuitable, one that was raised against *Manduca sexta* AST-C, which is almost identical to the crustacean peptide, proved suitable. This assay was highly specific for AST-C, as shown by HPLC-RIA of PO extracts (Fig. 6A), and showed very little cross-reactivity with AST-CC (Fig. 6B, inset).

Measurement of AST-C in SPE purified hemolymph samples showed that some AST-C was released at between stages E70 and E90 prior to ecdysis, but with a massive release (peaking at 800–100 fmol ml^−1^) exactly upon complete emergence from the old exoskeleton (E100). Within 2 h of ecdysis, levels diminished to basal values, becoming almost undetectable within 24 h (Fig. 6B). Simultaneous measurement of AST-C, CCAP and BURS in paired PO samples taken from post-molt, inter-molt, pre-molt and ecdysis crabs showed good pairwise correlations for all three hormones: Correlation coefficients were: AST-C/BURS, 0.73; AST-C/CCAP, 0.59; and CCAP/BURS, 0.77. PO contents for all were lowest in post-molt, increasing through inter-molt, and reaching a peak in late pre-molt (D2–3) before declining during ecdysis (Fig. 6C).

### Bioassays

**Fig. 7.**
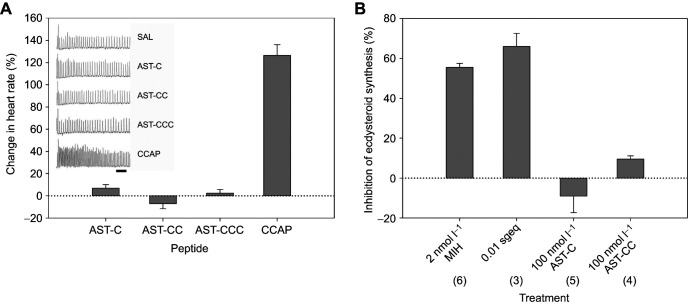
**AST-C peptides have no effect on ecdysteroid synthesis or heart rate.** (A,B) Bioassays to determine possible myoactive and/or inhibitory effects of AST-C, AST-CC and AST-CCC on semi-isolated heart preparations (A), and inhibition of ecdysteroid synthesis by Y-organs (B). (A) Heart preparations were recorded for 2 min after sequential addition of each peptide (10^−6^ mol l^−1^), as indicated. Four preparations were used. Data are means+s.e.m. Representative traces are shown. Bar indicates 10 s. (B) Y-organ bioassays were performed as indicated, to determine possible inhibitory effects of 100 nmol l^−1^ AST-C and AST-CC on ecdysteroid synthesis. Controls used native MIH (2 nmol l^−1^) and sinus gland extract (0.01 equivalents per Y-organ, which contained ∼0.7 nmol l^−1^ MIH and 7 nmol l^−1^ CHH). Data are means+s.e.m. *N*=3–6 as indicated.

Bioassays to investigate role of AST-C in the control of heart rate and ecdysteroid synthesis by Y-organs were performed in the light of recent research suggesting involvement of AST-C in these processes in crustaceans. Heartbeat frequency and amplitude were entirely unaffected by 10^−6^ mol l^−1^ application of AST-C, AST-CC and AST-CCC, whilst CCAP at this concentration more than doubled heart rate and increased amplitude. Incubation of YO with 100 nmol l^−1^ AST-C or AST-CC was entirely without effect on ecdysteroid synthesis, compared to the large inhibitory effect of 2 nmol l^−1^ molt inhibiting hormone or crude sinus gland extract (Fig. 7B).

## DISCUSSION

In this study, we have isolated and functionally confirmed the sequence encoding the AST-C and AST-CC receptor in *C. maenas*, and that it is activated by AST-C and AST-CC but not by AST-CCC. Expression patterns and complex neural architecture of the AST-C, AST-CC and AST-CCC peptide family, together with release patterns of AST-C and AST-CC, show that these peptides are co-released with CCAP and BURS during the completion of ecdysis. These findings revealed hitherto unexplored complexity in the hormone cascades occurring during this highly stereotyped behavioral sequence in crustaceans.

Although our previously sequenced neural transcriptome revealed four candidate receptors for the AST-C family peptides (Oliphant et al., 2018), only one (transcript 71151d) AST-CRd was activated equally by both AST-C and AST-CC (ED_50_ 2-6 nmol l^−1^). This transcript was not activated by AST-CCC, except for a slight activation at extremely high (10^−6^ mol l^−1^) doses. Thus, the cognate receptor for AST-CCC remains elusive. Despite the well-known similarity in receptor sequences between AST-CR and the somatostatin receptor (Krienkamp et al., 2002; Mayoral et al., 2010; Jékely, 2013; Verlinden et al., 2015; Urlacher et al., 2016), the vertebrate paralog somatostatin was without effect. Whilst dipterans (e.g. *Drosophila melanogaster* and *Aedes aegypti*) possess two AST-C receptors, in other insect orders (e.g. Coleoptera, Lepidoptera, Hymenoptera and Hemiptera), only one receptor is found (Krienkamp et al., 2002; Mayoral et al., 2010), and in the red flour beetle, *Tribolium castaneum*, the AST-C receptor is preferentially activated by AST-C (ED_50_ 25 nmol l^−1^) and AST-CC (ED_50_ 105.7 nmol l^−1^) (Audsley et al., 2013). For the honeybee, *Apis mellifera*, a single AST-C receptor is equally activated by both AST-C and AST-CC (EC_50_ values 14.6 and 15.4 nmol l^−1^, respectively) (Urlacher et al., 2016).

For decapod crustaceans, the only other AST-C receptors that have been functionally de-orphaned are those from *Cancer borealis*, using EGFP fused to the C-terminus of the AST-C receptor and expression in *Spodoptera frugiperda* SF9 cells (Dickinson et al., 2019), and from *H. americanus*, using chimeric EGFP constructs in *Tricoplusia ni* cells. In SF9 cells stably expressing either ASTCR1 or ASTCR2, TAMRA AST-C analogs localize to the plasma membrane, suggestive of receptor binding; of the four putative AST-C receptor transcripts, only two were activated by AST-C analogs in the cell expression assay described above (Muscato et al., 2021). In *S. paramamosain*, a CRE-Luc reporter assay in HEK293-transfected cells has been used to functionally de-orphan an AST-C receptor that was activated by AST-C (ED_50_ ∼6 nmol l^−1^) but not by AST-CCC (Liu et al., 2021). For the three crab species for which AST-CR has been functionally de-orphaned, the AST-C and AST-CC receptor shows very remarkable sequence identity, only 14/426 residues differ in the extracellular N- and intracellular C-terminal domains.

Regarding tissue distribution of the AST-C and AST-CC receptor, end-point PCR showed extremely low levels of expression, as expected. Apart from the CNS, the only other tissues exhibiting unambiguous expression were (mature) oocytes, the cardiac ganglion and hemocytes. This distribution bears some similarity with that reported for *S. paramamosain* (Liu et al., 2021) in that transcripts were observed in ovarian tissue, but were very different in other respects, particularly in expression in the CNS and in the wide distribution of transcripts in other tissues, which were not observed in our study.

Whilst our results did not show detectable AST-CR expression in the YO, which was also noted in our analysis of the *C. maenas* YO transcriptome (Oliphant et al., 2018), an analysis of much greater depth for *Gecarcinus lateralis* YO transcriptomes showed extremely low RPKM values throughout much of the molt cycle (Tran et al., 2019). For *S. paramamosain,* AST-C receptor transcripts were noted at moderate abundance in the YOs (Liu et al., 2021), and these authors have suggested that AST-C at extremely high, non-physiological, doses (10^−6^ M) can slightly inhibit ecdysteroid synthesis by YOs *in vitro*. We have been unable to repeat this result. Using a more discriminatory and sensitive RIA in which the antiserum (HB-2E) equally recognizes both the ecdysteroids synthesized by *C. maenas* (ecdysone and 25-deoxyecdysone), neither AST-C nor AST-CC inhibited ecdysteroid synthesis at high doses (100 nmol l^−1^) compared with full inhibition with MIH at a physiologically relevant dose (2 nmol l^−1^). Furthermore, in the Liu et al. (2021) study, an EIA which measured 20-hydroxyecdysone was used; this ecdysteroid is not secreted by portunid crab YOs but ecdysone and 25-deoxyecdysone is hydroxylated to 20-hydroxyecdysone and Ponasterone A in peripheral tissues (Mykles, 2011).

Liu et al. (2019) have reported that *in vivo* injection of AST-C inhibits vitellogenesis in *S. paramamosain*. It is likely that vitellogenesis takes place during inter-molt in this species, as it does in *C. maenas* (Styrishave et al., 2008), a closely related portunid crab. Since AST-C and likely AST-CC are only ephemerally released during the completion of ecdysis in the latter species, involvement of AST-C in inhibition of vitellogenesis seems entirely improbable, and it is extremely unlikely that *in vivo* injection of AST-C, as reported by Liu et al. (2019), has any biological relevance in normal physiology.

We detected AST-CR transcripts in cardiac ganglia, which has also been seen for *H. americanus* (Muscato et al., 2021). Application of AST-C or AST-CC in this species often results in a decrease in amplitude and frequency of heart rate, but also, in some individuals, in the converse, suggesting there are state-dependent responses (Dickinson et al., 2018). It has been proposed that these might in part be due to differential distribution of AST-CR1 in the cardiac ganglion. We perfused semi-isolated heart preparations of *C. maenas* with each peptide but found these to have no effect on heart rate or amplitude. Nevertheless, since only four preparations were used (with wash out between applications of each), it is possible that modulation might have occurred only infrequently due to differential expression of AST-CR, as suggested by Dickinson et al. (2018). However, for other modulatory peptides used in our heart assays, e.g. DH-31 proctolin and CCAP (Alexander et al., 2018), responses were observed in every preparation. It would clearly be worthwhile to determine mRNA levels encoding the AST-C and AST-CC receptors in many more preparations to determine this interesting hypothesis by qRT-PCR.

Moderate levels of transcripts encoding AST-CR were observed in hemocyte preparations. Whilst this was not seen in *S. paramamosain* (Liu et al., 2019), our findings are of interest, since AST-C modulates nociception and immunity in *Drosophila* (Bachtel et al., 2018), and, as detailed later, the highest levels of circulating AST-C are only found on completion of exuviation (E100). An attractive hypothesis would be that, at this time, when the new cuticle is vulnerable to pathogen invasion, a similar downregulation of components of the innate immune system might prevent immunopathology or reduce unnecessary metabolic cost after microbial stimulation, as suggested previously (Bachtel et al., 2018). In contrast, it has been proposed that in hemocytes of *S. paramamosain* AST-B and AST-BR together increase expression of a variety of immune effector molecules and components of the NO signaling pathway (Xu et al., 2021). Clearly, further investigation into the interactions of AST peptides with the immune system are now timely; a recent transcriptomic study on immune component dynamics in *Portunus trituberculatus* has revealed a substantial number (98) of genes involved in immunity that are differentially expressed during the molt cycle (Liu et al., 2022).

Using a combination of whole-mount IHC, conventional ISH and HCR–FISH, we have shown that, in the ventral ganglion, a set of neurons known to express CCAP and BURS (Webster et al., 2013) also expresses AST-C peptides; by using HCR-FISH in dual labeling experiments, transcripts for both AST-C and AST-CC were found colocalized in neurons in the abdominal ganglia. Thus, all these neurons that form the well-established anatomy of the large type-1 (cdn) and small type-2 (cdc) neurons (Dircksen, 1998) synthesize a cocktail of at least four neuropeptides; IHC of the release site for axon terminations of these neurons – the POs – clearly show that all four peptides are present in all the dendrites and secretory boutons. Since the release dynamics of CCAP and BURS during the ecdysis program are known in some detail, it was unsurprising that these were mirrored exactly for AST-C, in terms of circulating titers during the ecdysis program and correlations between the quantities of CCAP, BURS and AST-C in the POs during the molt cycle. When comparing the decline in AST-C during immediate post-molt, levels fell to around 20% of the peak value at E100 within 1 h of completion of ecdysis. This would suggest that the half-time in circulation was like that of CCAP, but longer than for BURS, where levels decline to about 5% of the maximum within 1 h of completion of ecdysis (Webster et al., 2013).

AST-C, AST-CC and AST-CCC immunopositive structures in the other regions of the CNS examined were complex and often difficult to determine. In the CG, only AST-C and AST-CCC were expressed in several neurons in the anterior medial cell group (nomenclature according to Sandeman et al., 1992) and they were never co-expressed in the same neurons. Interestingly, large groups of hundreds of small (10–15 µm) cell bodies were seen in the dorsal lateral cell group where mRNA expression was observed; these neurons were not observed by IHC, suggesting low translation levels. Nevertheless, dual-labeling HCR–FISH, which is much more sensitive than conventional ISH, showed that expression of AST-C and AST-CCC transcripts were non overlapping. In *S. paramamosain*, perikarya expressing AST-C have been observed in broadly similar areas of the CG by ISH of sections and by whole-mount IHC (Liu et al., 2019), but were difficult to compare with the results obtained here. AST-C-immunopositive structures have been detailed in the copepod (*Calanus finmarchicus*) CNS (Wilson and Christie, 2010), but the dissimilar morphology of the nervous system in maxillopodan crustaceans and malacostracans makes direct comparison difficult, if not impossible. When comparing the CG nervous system with that of the VG, it is notable that, in the CG, only AST-C and AST-CCC are expressed, and these neurons would have neurotransmitter and/or modulatory roles; but in the VG, only AST-C and AST-CC are expressed, and these have neurohormonal roles. However, we did observe some very weak hybridization signals for AST-CCC in cells surrounding the sternal artery, but could not definitively identify these via HCR–FISH. They were buried deep in the neuropil and consequently could not be visualized via IHC, given the size and thickness of this tissue. We did not detect AST-C and AST-CC immunopositive neurons in the eyestalk or hindgut. This last observation was surprising because tissue specific transcriptomes from *C. maenas* show that AST-C but not AST-CCC transcripts are present in the gut (Verbruggen et al., 2015). However, the three AST-C transcripts are widely distributed in many tissues, but only AST-CC is exclusively present in the CNS. Clearly, further studies are now needed to confirm whether these tissues also contain translated peptides, but the caveat here is that the exceptionally low levels of expression seen by comparing transcriptome RPKM in various tissues, which may be more discriminatory and/or sensitive with respect to end-point PCR, might be misleading if transcription is not mirrored by concomitant translation.

For the stomatogastric nervous system, a limited study was performed. Descending axons from the CG immunolabeled for both AST C and AST-CC or AST-CCC formed extensive arborizations in the COG, and AST C and AST-CC exited via the superior esophageal nerve (son) and inferior esophageal nerve (ion). No obvious cell bodies were noted in the COG for AST-C and AST-CC, but for AST-CCC, three large perikarya were observed. These results (although preliminary) contrast with studies using the same antisera in *H. americanus* (Dickinson et al., 2019), where approximately 14 AST-C and AST-CC somata were seen in each COG and 42 somata immunopositive for AST-CCC were also seen in the COG. No axons containing AST-CCC were seen exiting via the ion, whereas, in this study, several were seen. Curiously, axons labeled for AST-CCC were never seen in the ion or son, but axons for both were observed in the stomatogastric nerve. It is probable that these (AST-CCC) were not well labeled in our preparations but the possibility, albeit remote, exists that innervation of AST-CCC-containing axons was via the esophageal ganglion and stomatogastric nerve.

For *H. americanus*, two large perikarya immunopositive for AST-CCC were observed (Dickinson et al., 2019), but in our *C. maenas* preparations none were seen. However, 28 small (∼10 µm) AST-C and AST-CC immunopositive cells, without any visible axons were seen at the periphery of the STG. These were clearly from the much larger motor neurons of the STG, the connectome of which is well known (reviewed by Marder, 2012) and some of which, in *C. maenas*, express DH-31 (Alexander et al., 2018). It is tempting to speculate that these were glial cells, and, in this context, it is interesting to note that the vertebrate AST-C paralog, somatostatin, is well known to be expressed in this cell type in the mammalian pituitary and hypothalamus (Davidson and Gillies, 1993; Chronwall et al., 2000). However, positive identification of these cells, using glial-specific markers, as used to identify glia in crustaceans (Wajsenzon et al., 2016), should be performed to confirm these results.

This study has shown that a combined approach of receptor de-orphanization coupled with cognate gene and peptide expression studies, at the molecular, cellular and organismal levels, is a valuable strategy in functional neuroendocrinology. For peptides in the AST-C family, we can conclude that a single receptor is activated (equally) by AST-C and AST-CC, but that the ligand–receptor pair for AST-CCC remains an orphan. AST-C and AST-CCC have neurotransmitter and/or modulatory roles in the CG and STG nervous systems, but AST-C and AST-CC act as neurohormones that are released for a very short time from neurons in the VG, which also simultaneously release CCAP and BURS, on completion of ecdysis. Whilst the functions of the latter hormones in the ecdysis program are well known, the function(s) of AST-C and AST-CC have yet to be established. However, the expression of the AST-C and AST-CC receptor in hemocytes is strongly suggestive of involvement in immune system responses. Our findings may thus stimulate further research into the neuroendocrine–immune axis in crustaceans.

## Supplementary Material

10.1242/jexbio.249929_sup1 Supplementary information

## References

[JEB249929C1] Alexander, J., Oliphant, A., Wilcockson, D. C. and Webster, S. G. (2018). Functional identification and characterization of the diuretic hormone 31 (DH31) signaling system in the green shore crab, *Carcinus maenas*. *Front. Neurosci.* 12, 454. 10.3389/fnins.2018.00454 30022930 PMC6039563

[JEB249929C2] Audsley, N., Vandersmissen, H. P., Weaver, R., Dani, P., Matthews, J., Down, R., Vuerinckx, K., Kim, Y.-J. and Vanden Broek, J. (2013). Characterization and tissue distribution of the PISCF allatostatin receptor in the red flour beetle, *Tribolium castaneum*. *Insect Biochem. Mol. Biol.* 43, 65-74. 10.1016/j.ibmb.2012.09.007 23085356

[JEB249929C3] Bachtel, N. D., Hovsepian, G. A., Nixon, D. F. and Eleftherianos, I. (2018). Allatostatin C modulates nociception and immunity in *Drosophila*. *Sci. Rep.* 8, 7501. 10.1038/s41598-018-25855-1 29760446 PMC5951828

[JEB249929C4] Bao, C., Yang, Y., Zeng, C., Huang, H. and Ye, H. (2018). Identifying neuropeptide GPCRs in the mud crab, *Scylla paramamosain*, by combinatorial bioinformatics analysis. *Gen. Comp. Endocrinol.* 269, 122-130. 10.1016/j.ygcen.2018.09.002 30189191

[JEB249929C5] Bauknecht, P. and Jékely, G. (2015). Large-scale combinatorial deorphanization of *Platynereis* neuropeptide GPCRs. *Cell Rep.* 12, 684-693. 10.1016/j.celrep.2015.06.052 26190115

[JEB249929C6] Bendena, W. G. and Tobe, S. S. (2012). Families of allatoregulator sequences: a 2011 perspective. *Can. J. Zool.* 90, 521-544. 10.1139/z2012-012

[JEB249929C7] Bolton, A. E. (1989). Comparative methods for the radiolabelling of peptides. In *Neuroendocrine Peptide Methodology* (ed. P.M. Conn), pp. 391-401. New York, London: Academic Press.

[JEB249929C8] Bonasio, R., Zhang, G., Ye, C., Mutti, N. S., Fang, X., Qin, N., Donahue, G., Yang, P., Li, Q., Li, C. et al. (2010). Genomic comparison of the ants *Camponotus floridanus* and *Harpegnathos saltator*. *Science* 329, 1068-1071. 10.1126/science.1192428 20798317 PMC3772619

[JEB249929C9] Bruce, H. S., Jerz, G., Kelly, S., McCarthy, J., Pomerantz, A., Senevirathne, G., Sherrad, A., Sun, D. A., Wolff, C. and Patel, N. H. (2021). Hybridization chain Reaction (HCR) in Situ Protocol. 10.17504/protocols.io.bunznvf6.

[JEB249929C10] Choi, H. M. T., Schwarzkopf, M., Fornace, M. E., Acharya, A., Artavanis, G., Stegmaier, A. and Pierce, N. A. (2018). Third generation *in situ* hybridization chain reaction: multiplexed, quantitative, sensitive, versatile, robust. *Development* 145, dev165753. 10.1242/dev.165753 29945988 PMC6031405

[JEB249929C11] Christie, A. E. (2016). Expansion of the neuropeptidome of the globally invasive marine crab *Carcinus maenas*. *Gen. Comp. Endocrinol.* 235, 150-169. 10.1016/j.ygcen.2016.05.013 27179880

[JEB249929C12] Chronwall, B. M., Sands, S. A., Cummings, K. C. III and Schwartz, J. P. (2000). Glial somatostatin-14 expression in the rat pituitary intermediate lobe: a possible neurotrophic function during development? *Int. J. Dev. Neurosci.* 18, 685-692. 10.1016/S0736-5748(00)00035-6 10978847

[JEB249929C13] Davidson, K. and Gillies, G. E. (1993). Neuronal vs. glial somatostatin in the hypothalamus: a cell culture study of the ontogenesis of cellular location, content and release. *Brain Res.* 624, 75-84. 10.1016/0006-8993(93)90062-R 7902774

[JEB249929C14] Dickinson, P. S., Armstrong, M. K., Dickinson, E. S., Fernandez, R., Miller, A., Pong, S., Powers, B. W., Pupo-Wiss, A., Stanhope, M. E., Walsh, P. J. et al. (2018). Three members of a peptide family are differentially distributed and elicit differential state-dependent responses in a pattern generator-effector system. *J. Neurophysiol.* 119, 1767-1781. 10.1152/jn.00850.2017 29384453 PMC6008092

[JEB249929C15] Dickinson, P. S., Hull, J. J., Miller, A., Oleisky, E. R. and Christie, A. E. (2019). To what extent may receptor gene diversity/complement contribute to functional flexibility in a simple pattern generating neural network? *Comp. Biochem. Physiol. D* 30, 262-282. 10.1016/j.cbd.2019.03.002 PMC7080212 30974344

[JEB249929C16] Dircksen, H. (1998). Conserved crustacean cardioactive peptide (CCAP) neuronal networks and functions in arthropod evolution. In *Recent Advances in Arthropod Endocrinology*. Society for Experimental Biology seminar series: 65 (ed. G. M. Coast and S. G. Webster), pp. 302-333. Cambridge: Cambridge University Press.

[JEB249929C17] Dircksen, H., Neupert, S., Predel, R., Verleyen, P., Huybrechts, J., Strauss, J., Hauser, F., Stafflinger, E., Schneider, M., Pauwels, J. et al. (2011). Genomics, transcriptomics, and peptidomics of *Daphnia pulex* neuropeptides and protein hormones. *J. Proteome Res.* 10, 4478-4504. 10.1021/pr200284e 21830762

[JEB249929C18] Drach, P. and Tchernigovtzeff, C. (1967). Sur la méthode de determination des stades d'intermue et son application Générale aux crustacés. *Vie Milieu* 18, 595-610.

[JEB249929C19] Duve, H., Wren, P. and Thorpe, A. (1995). Innervation of the foregut of the cockroach *Leucophaea maderae* and inhibition of spontaneous contractile activity by callatostatin neuropeptides. *Physiol. Entomol.* 20, 33-44. 10.1111/j.1365-3032.1995.tb00798.x

[JEB249929C20] Duve, H., Johnsen, A. H., Maestro, J. L., Scott, A. G., Jaros, P. P. and Thorpe, A. (1997). Isolation and identification of multiple neuropeptides of the allatostatin superfamily in the shore crab *Carcinus maenas*. *Eur. J. Biochem.* 250, 727-734. 10.1111/j.1432-1033.1997.00727.x 9461295

[JEB249929C21] Fusé, M., Zhang, J. R., Partridge, E., Nachman, R. J., Orchard, I., Bendena, W. G. and Tobe, S. S. (1999). Effects of an allatostatin and a myosuppressin on midgut carbohydrate enzyme activity in the cockroach *Diploptera punctata*. *Peptides* 20, 1285-1293. 10.1016/S0196-9781(99)00133-3 10612442

[JEB249929C22] Hou, L., Jiang, F., Yang, P., Wang, X. and Kang, L. (2015). Molecular characterization and expression profiles of neuropeptide precursors in the migratory locust. *Insect Biochem. Mol. Biol.* 63, 63-71. 10.1016/j.ibmb.2015.05.014 26036749

[JEB249929C23] Hua, Y.-J., Tanaka, Y., Nakamura, K., Sakakibara, M., Nagata, S. and Katakoa, H. (1999). Identification of a prothoracicostatic peptide in the larval brain of the silkworm, *Bombyx mori*. *J. Biol. Chem.* 274, 31169-31173. 10.1074/jbc.274.44.31169 10531308

[JEB249929C24] Jékely, G. (2013). Global view of the evolution and diversity of metazoan neuropeptide signaling. *Proc. Natl. Acad. Sci. USA* 110, 8702-8707. 10.1073/pnas.1221833110 23637342 PMC3666674

[JEB249929C25] Kearse, M., Moir, R., Wilson, A., Stones-Havas, S., Cheung, M., Sturrock, S., Buxton, S., Cooper, A., Markowitz, S., Duran, C. et al. (2012). Geneious Basic: an integrated and extendable desktop software platform for the organization and analysis of sequence data. *Bioinformatics* 28, 1647-1649. 10.1093/bioinformatics/bts199 22543367 PMC3371832

[JEB249929C26] Kim, Y.-J., Žitňan, D., Cho, K.-H., Schooley, D. A., Mizoguchi, A. and Adams, M. E. (2006). Central peptidergic ensembles associated with organization of an innate behavior. *Proc. Natl. Acad. Sci. USA* 103, 14211-14216. 10.1073/pnas.0603459103 16968777 PMC1599936

[JEB249929C27] Kolodziejczyk, A. and Nässel, D. R. (2011). A novel wide-field neuron with branches in the lamina of the *Drosophila* visual system expresses myoinhibitory peptide and may be associated with the clock. *Cell Tissue Res.* 343, 357-369. 10.1007/s00441-010-1100-7 21174124

[JEB249929C28] Kramer, S. J., Toschi, A., Miller, C. A., Katakoa, H., Quistad, G. B., Li, J. P., Carney, R. L. and Schooley, D. A. (1991). Identification of an allatostatin from the tobacco hornworm Manduca sexta. *Proc. Natl. Acad Sci. USA* 88, 9458-9462. 10.1073/pnas.88.21.9458 1946359 PMC52737

[JEB249929C29] Krienkamp, H.-J., Larusson, H. J., Witte, I., Roeder, T., Birgül, N., Hönck, H.-H., Harder, S., Ellinghausen, G., Buck, F., Ellinghausen, G. et al. (2002). Functional annotation of two orphan G-protein coupled receptors, Drostar 1 and -2 from *Drosophila melanogaster* and their ligands by reverse pharmacology. *J. Biol. Chem* 277, 39937-39943. 10.1074/jbc.M206931200 12167655

[JEB249929C30] Lange, A. B., Bendena, W. G. and Tobe, S. S. (1995). The effect of thirteen Dip-allatostatins on myogenic and induced contractions of the cockroach *(Diploptera punctata*) hindgut. *J. Insect Physiol.* 41, 581-588. 10.1016/0022-1910(95)00008-I

[JEB249929C31] Lange, A. B., Alim, U., Vandersmissen, H. P., Mizoguchi, A., Vanden Broeck, J. and Orchard, I. (2012). The distribution and physiological effects of the myoinhibiting peptides in the kissing bug, *Rhodnius prolixus*. *Front. Neurosci.* 6, 98. 10.3389/fnins.2012.00098 22783161 PMC3390896

[JEB249929C32] Liu, A., Liu, F., Shi, W., Huang, H., Wang, G. and Ye, H. (2019). C-type allatostatin and its putative receptor from the mud crab serve an inhibitory role in ovarian development. *J. Exp. Biol.* 222, jeb207985. 10.1242/jeb.207985 31558587

[JEB249929C33] Liu, A., Shi, W., Liu, D. and Ye, H. (2021). A possible role of allatostatin C in inhibiting ecdysone biosynthesis revealed in the mud crab *Scylla paramamosain.* *Front. Mar. Sci.* 8, 740251. 10.3389/fmars.2021.740251

[JEB249929C34] Liu, M., Ni, H., Zhang, X., Sun, Q., Wu, X. and He, J. (2022). Comparative transcriptomics reveals the immune dynamics during the molting cycle of swimming crab *Portunus trituberculatus*. *Front. Immunol.* 13, 1037739. 10.3389/fimmu.2022.1037739 36389847 PMC9659622

[JEB249929C35] Lorenz, M. W., Kellner, R. and Hoffmann, K. H. (1995). A family of neuropeptides that inhibit juvenile hormone biosynthesis in the cricket, *Gryllus bimaculatus*. *J. Biol. Chem.* 270, 21103-21108. 10.1074/jbc.270.36.21103 7673141

[JEB249929C36] Ma, M., Bors, E. K., Dickinson, E. S., Kwiatkowski, M. A., Sousa, G. L., Henry, R. P., Smith, C. M., Towle, D. W., Christie, A. E. and Li, L. (2009). Characterization of the *Carcinus maenas* neuropeptidome by mass spectrometry and functional genomics. *Gen. Comp. Endocrinol.* 161, 320-334. 10.1016/j.ygcen.2009.01.015 19523386 PMC2888039

[JEB249929C37] Marder, E. (2012). Neuromodulation of neuronal circuits: back to the future. *Neuron* 76, 1-11. 10.1016/j.neuron.2012.09.010 23040802 PMC3482119

[JEB249929C38] Martin, D., Piulachs, M. D. and Bellés, X. (1996). Inhibition of vitellogenesis production by allatostatin in the German cockroach. *Mol. Cell. Endocrinol.* 121, 191-196. 10.1016/0303-7207(96)03864-6 8892320

[JEB249929C39] Matthews, H. J., Audsley, N. and Weaver, R. J. (2007). Interactions between allatostatins and allatotropin on spontaneous contractions of the foregut of larval *Lacanobia oleracea*. *J. Insect Physiol.* 53, 75-83. 10.1016/j.jinsphys.2006.10.007 17150228

[JEB249929C40] Mayoral, J. G., Nouzova, M., Brockhoff, A., Goodwin, M., Hernandez-Martinez, S., Richter, D., Meyerhof, W. and Noriega, F. G. (2010). Allatostatin-C receptors in mosquitoes. *Peptides* 31, 442-450. 10.1016/j.peptides.2009.04.013 19409436 PMC2826609

[JEB249929C41] Muscato, A. J., Walsh, P., Pong, S., Pupo, A., Gross, R. J., Christie, A. E., Hull, J. J. and Dickinson, P. S. (2021). Does differential receptor distribution underlie variable responses to a neuropeptide in the lobster cardiac system? *Int. J. Mol. Sci.* 22, 8703. 10.3390/ijms22168703 34445418 PMC8395929

[JEB249929C42] Mykles, D. L. (2011). Ecdysteroid metabolism in crustaceans. *J. Steroid Biochem. Mol. Biol.* 127, 196-203. 10.1016/j.jsbmb.2010.09.001 20837145

[JEB249929C43] Oliphant, A., Alexander, J. L., Swain, M. T., Webster, S. G. and Wilcockson, D. C. (2018). Transcriptomic analysis of crustacean neuropeptide signaling during the moult cycle of the green shore crab, *Carcinus maenas*. *BMC Genomics* 19, 711. 10.1186/s12864-018-5057-3 30257651 PMC6158917

[JEB249929C44] Phlippen, M. K., Webster, S. G., Chung, J. S. and Dircksen, H. (2000). Ecdysis of decapod crustaceans is associated with a dramatic release of crustacean cardioactive peptide into the haemolymph. *J. Exp. Biol.* 203, 521-536. 10.1242/jeb.203.3.521 10637181

[JEB249929C45] Pratt, G. E., Farnsworth, D. E., Siegel, N. R., Fok, K. F. and Feyereisen, R. (1989). Identification of an allatostatin from adult *Diploptera punctata*. *Biochem. Biophys. Res. Commun.* 163, 1243-1247. 10.1016/0006-291X(89)91111-X 2783135

[JEB249929C46] Sandeman, D., Sandeman, R., Derby, C. and Schmidt, M. (1992). Morphology of the brain of crayfish, crabs and spiny lobsters: a common nomenclature for homologous structures. *Biol. Bull.* 183, 304-326. 10.2307/1542217 29300672

[JEB249929C47] Saver, M. A., Wilkens, J. L. and Syed, N. I. (1999). *In situ* and *in vitro* identification and characterization of cardiac ganglion neurons in the crab, *Carcinus maenas*. *J. Neurophysiol.* 81, 2964-2976. 10.1152/jn.1999.81.6.2964 10368413

[JEB249929C48] Schoofs, L., Holman, G. M., Hayes, T. K., Nachman, R. J. and De Loof, A. (1991). Isolation, identification and synthesis of locustamyoinhibiting peptide (LOM-MIP), a novel biologically active neuropeptide from *Locusta migratoria*. *Reg. Peptides* 36, 111-119. 10.1016/0167-0115(91)90199-Q 1796179

[JEB249929C49] Schulze, J., Neupert, S., Schmidt, L., Predel, R., Lamkemeyer, T., Homberg, U. and Stengl, M. (2012). Myoinhibitory peptides in the brain of the cockroach *Leucophaea maderae* and colocalization with pigment-dispersing factor in circadian pacemaker cells. *J. Comp. Neurol.* 520, 1078-1097. 10.1002/cne.22785 22095637

[JEB249929C50] Stay, B. and Tobe, S. S. (2007). The role of allatostatins in juvenile hormone synthesis in insects and crustaceans. *Annu. Rev. Entomol.* 52, 277-299. 10.1146/annurev.ento.51.110104.151050 16968202

[JEB249929C51] Stefanini, M., De Martino, C. and Zamboni, L. (1967). Fixation of ejaculated spermatozoa for electron microscopy. *Nature* 216, 173-174. 10.1038/216173a0 4862079

[JEB249929C52] Styrishave, B., Lund, T. and Andersen, O. (2008). Ecdysteroids in female shore crabs *Carcinus maenas* during the moulting cycle and oocyte development. *J. Mar. Biol. Assn. UK* 88, 575-581. 10.1017/S0025315408000878

[JEB249929C53] Szabo, T. M., Chen, R., Goeritz, M. L., Maloney, R. T., Tang, L. S., Li, L. and Marder, E. (2011). Distribution and physiological effects of B-type allatostatins (Myoinhibitory peptides, MIPs) in the stomatogastric nervous system of the crab *Cancer borealis*. *J.Comp. Neurol.* 519, 2658-2676. 10.1002/cne.22654 21491432 PMC3245975

[JEB249929C54] Tran, N. M., Mykles, D. L., Elizur, A. and Ventura, T. (2019). Characterization of G-protein coupled receptors from the blackback land crab *Gecarcinus lateralis* Y organ transcriptome over the molt cycle. *BMC Genomics* 20, 74. 10.1186/s12864-018-5363-9 30669976 PMC6341585

[JEB249929C55] Urlacher, E., Soustelle, L., Parmentier, M.-L., Verlinden, H., Gherardi, M.-J., Fourmy, D., Mercer, A. R., Devaud, J.-M. and Massou, I. (2016). Honey bee allatostatins target Galanin/Somatostatin-like receptors and modulate learning: a conserved function? *PLoS ONE* 11, e0146248. 10.1371/journal.pone.0146248 26741132 PMC4704819

[JEB249929C56] Veenstra, J. A. (2016a). Similarities between decapod and insect neuropeptidomes. *PeerJ* 4, e2043. 10.7717/peerj.2043 27257538 PMC4888303

[JEB249929C57] Veenstra, J. A. (2016b). Neuropeptide evolution: Chelicerate neurohormone and neuropeptide genes may reflect one or more whole genome duplications. *Gen. Comp. Endocrinol.* 229, 41-55. 10.1016/j.ygcen.2015.11.019 26928473

[JEB249929C58] Veenstra, J. A. (2016c). Allatostatins C, double C and triple C, the result of a local gene triplication in an ancestral arthropod. *Gen. Comp. Endocrinol.* 230-231, 153-157. 10.1016/j.ygcen.2016.04.013 27102937

[JEB249929C59] Verbruggen, B., Bickley, L. K., Santos, E. M., Tyler, C. R., Stentiford, G. D., Bateman, K. S. and Van Aerle, R. (2015). *De novo* assembly of the *Carcinus maenas* transcriptome and characterization of innate immune system pathways. *BMC Genomics* 16, 458. 10.1186/s12864-015-1667-1 26076827 PMC4469326

[JEB249929C60] Verlinden, H., Gijbels, M., Lismont, E., Lenaerts, C., Vanden Broeck, J. and Marchal, E. (2015). The pleiotropic allatoregulatory neuropeptides and their receptors: a mini-review. *J. Insect Physiol.* 80, 2-14. 10.1016/j.jinsphys.2015.04.004 25982521

[JEB249929C61] Vilaplana, L., Maestro, J. L., Piulachs, M.-D. and Bellés, X. (1999). Modulation of cardiac rhythm by allatostatins in the cockroach *Blatella germanica* (L.) (Dictyoptera, Blatellidae). *J. Insect Physiol.* 45, 1057-1064. 10.1016/S0022-1910(99)00089-X 12770266

[JEB249929C62] Wajsenzon, I. J. R., de Carvalho, L. A., Biancalana, A., da Silva, W. A. B., dos Santos Mermelstein, C., de Araujo, E. G. and Allodi, S. (2016). Culture of neural cells of the eyestalk of a mangrove crab is optimized on poly-L-ornithine substrate. *Cytotechnology* 68, 2193-2206. 10.1007/s10616-015-9942-1 26779908 PMC5023563

[JEB249929C63] Webster, S. G. (1986). Neurohormonal control of ecdysteroid biosynthesis by *Carcinus maenas* Y-Organs *in vitro*, and preliminary characterization of the putative molt-inhibiting hormone (MIH). *Gen. Comp. Endocrinol.* 61, 237-247. 10.1016/0016-6480(86)90201-7 3956985

[JEB249929C64] Webster, S. G., Wilcockson, D. C., Mrinalini, and Sharp, J. H. (2013). Bursicon and neuropeptide cascades during the ecdysis program of the shore crab, *Carcinus maenas*. *Gen. Comp. Endocrinol* 182, 54-64. 10.1016/j.ygcen.2012.11.018 23247273

[JEB249929C65] Wilcockson, D. C., Zhang, L., Hastings, M. H., Kyriacou, C. P. and Webster, S. G. (2011). A novel form of pigment-dispersing hormone in the central nervous system of the intertidal marine isopod, *Eurydice pulchra* (Leach). *J. Comp. Neurol.* 519, 562-575. 10.1002/cne.22533 21192084

[JEB249929C66] Wilson, C. H. and Christie, A. E. (2010). Distribution of C-type allatostatin (C-AST)-like immunoreactivity in the central nervous system of the copepod *Calanus finmarchicus*. *Gen. Comp. Endocrinol.* 167, 252-260. 10.1016/j.ygcen.2010.03.012 20338176 PMC2921218

[JEB249929C67] Xu, Z., Wei, Y., Wang, G. and Ye, H. (2021). B-type allatostatin regulates immune response of hemocytes in mud crab *Scylla paramamosain*. *Dev. Comp. Immunol.* 120, 104050. 10.1016/j.dci.2021.104050 33631272

[JEB249929C68] Yamanaka, N., Yamamoto, S., Žitňan, D., Watanabe, K., Kawada, T., Satake, H., Kaneko, Y., Hiruma, K., Tanaka, Y., Shinoda, T. et al. (2008). Neuropeptide receptor transcriptome reveals unidentified neuroendocrine pathways. *PLoS ONE* 3, e3048. 10.1371/journal.pone.0003048 18725956 PMC2516173

[JEB249929C69] Zhang, Y., Yañez Guerra, L. A., Egertová, M., Zampronio, C. G., Jones, A. M. and Elphick, M. R. (2020). Molecular and functional characterization of somatostatin-type signalling in a deuterostome invertebrate. *Open Biol.* 10, 200172. 10.1098/rsob.200172 32898470 PMC7536072

