# Supplementary material for: Allatostatin-C signaling in the crab Carcinus maenas is implicated in the ecdysis program
Source: J Exp Biol. 2025 Mar 17;228(5):JEB249929. doi: 10.1242/jeb.249929 (PMC11959706; doi:10.1242/jeb.249929)
Supplement: Supplementary information [file jexbio-228-249929-s1.pdf]

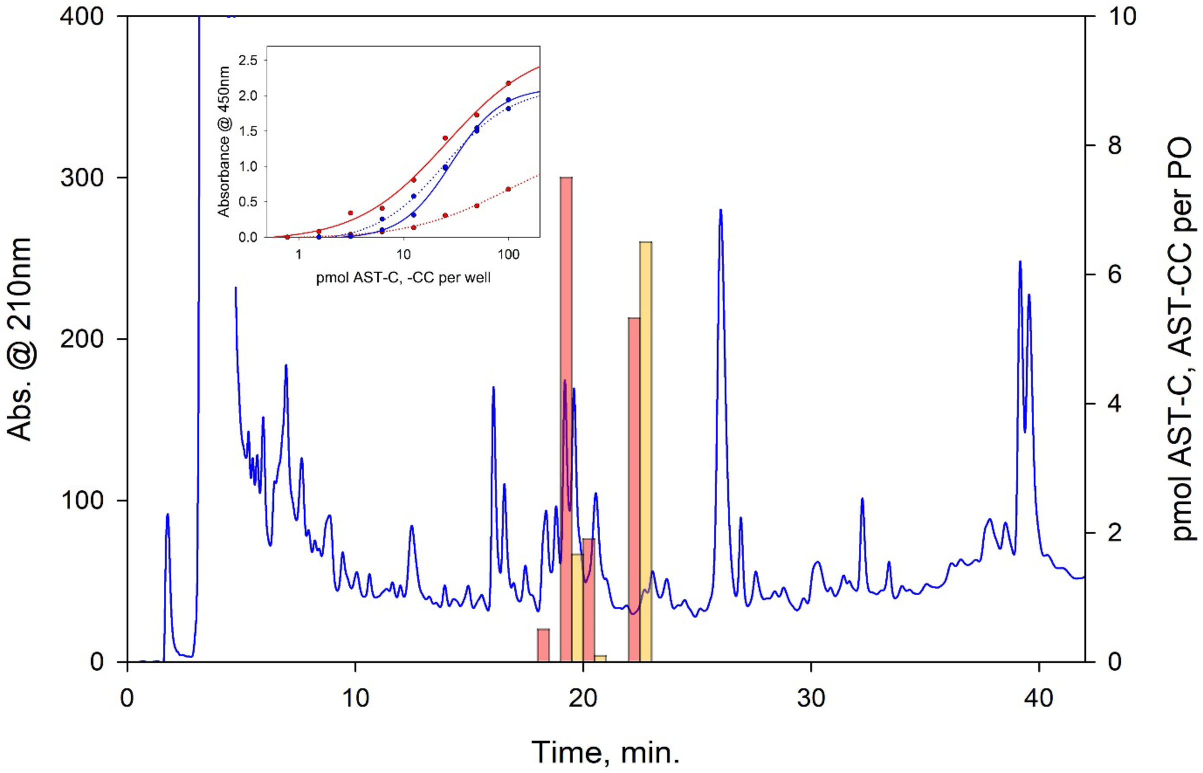

Fig. S1. HPLC-EIA of 12 *C. maenas* pericardial organs. Chromatographic conditions and EIA as described in the text. Retention times of AST-C and -CC are shown above bars. Pink bars: anti-AST-CC (Code 1262:1/8000), yellow bars anti-AST-C (Christie et al 2018:1/4000). Inset shows standard curves in the direct EIA. Solid red AST-CC dotted red AST-C: 1262 antiserum, Solid blue AST-C, dotted blue AST-CC Christie antiserum.

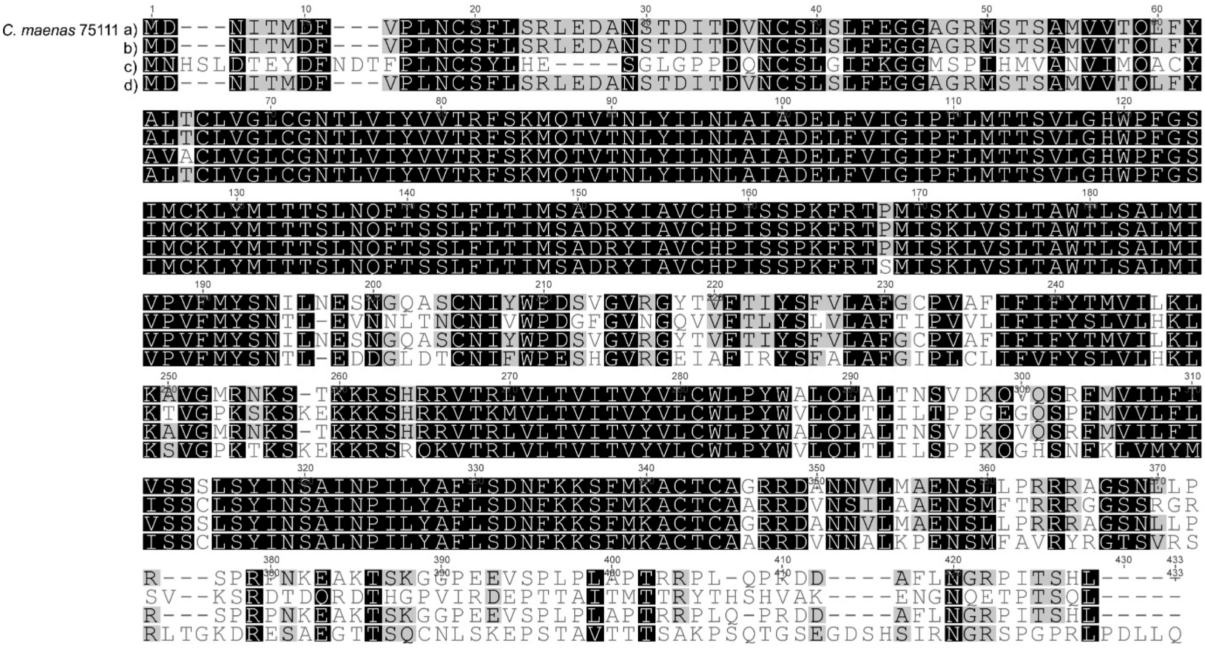

Fig. S2. Amino acid sequences of four putative AST-CR candidates. Identification codes as detailed in Oliphant et al. (2018).

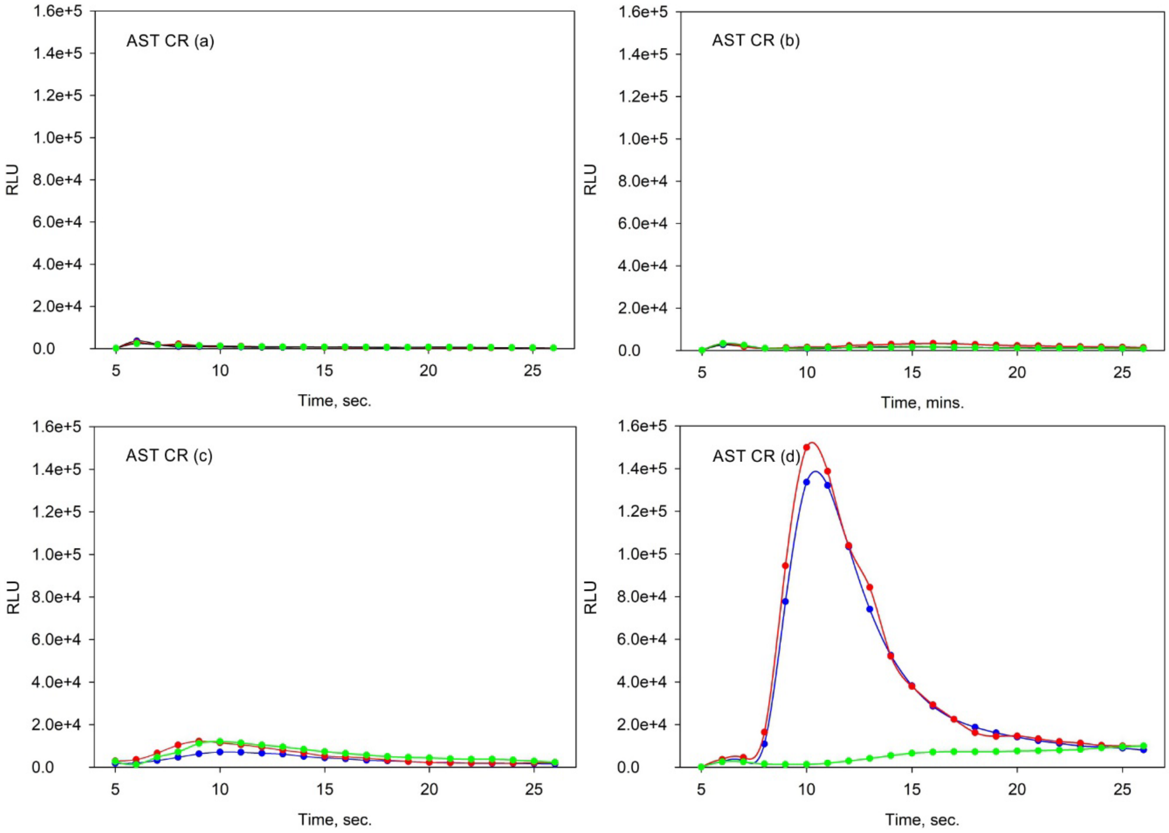

Fig. S3. Luminescent responses of CHO-K1-Aeq-Gα cells transiently expressing each of the putative AST-C receptors identified from neural transcriptomes of *C. maenas* (Oliphant *et al.* 2018) after exposure to 1μM of AST-C ((blue lines), AST-CC (redlines) and AST-CCC (green lines). Mean relative luminescence values (RLU) of quadruplicated samples were normalised to account for differences in luminescence (cell number) after lysis.

Table S1. Sequences of primers used for PCR and production of ISH probes for AST-C, -CC and -CCC.

**Name**	**Sequence 5'-3'**	**Tm**
Ast-C 41 F	TGGTATTGGCCCTCACTCAC	59.4
Ast-C 407 R	CACTGATGGTACCGAATCTGTC	58.6
Ast-C 41 F T7	TAA TAC GAC TCA CTA TAG GGTGGTATTGGCCCTCACTCAC	77.3
Ast-C 407 R	TAA TAC GAC TCA CTA TAG GGCACTGATGGTACCGAATCTGTC	77.9
Ast-CCa 11 F	CGGAAGGTCTCTCTACACTCAC	59.6
Ast-CCa 277 R	GATCTCTTGGCTCCTGTTGTAC	58.5
Ast-CCa 11 F T7	TAATACGACTCACTATAGGGCGGAAGGTCTCTCTACACTCAC	77.1
Ast-CCa 277 R T7	TAATACGACTCACTATAGGGGATCTCTTGGCTCCTGTTGTAC	76.9
Ast-CCb 29 F	CCAACATCCCGCTCATGATTAG	59.2
Ast-CCb 294 R	GAAGTAACACCTCCAGTACAGC	58.4
Ast-CCb 29 F T7	TAATACGACTCACTATAGGGCCAACATCCCGCTCATGATTAG	79.8
Ast-CCb 294 R T7	TAATACGACTCACTATAGGGGAAGTAACACCTCCAGTACAGC	75.7
Ast-CCC 1 F	ATGGTGTTCCCTCGTCACTG	59.7
Ast-CCC 343 R	ACCCTCTTTACTTCCTCTTGCC	59.7
Ast-CCC 1 F T7	TAATACGACTCACTATAGGGATGGTGTTCCCTCGTCACTG	78.1
Ast-CCC 343 R T7	TAATACGACTCACTATAGGGACCCTCTTTACTTCCTCTTGCC	76.9

Table S2. Sequences of peptides used in AST-C receptor deorphaning.

Peptide	Sequence
AST-C	pQIRYHQCYFNPISCF
AST-CC	GNGDGRLYWRCYFNAVSCF
AST-CCC	SYWKQCAFNAVSCF-NH_2
Somatostatin	AGCKNFFWKTFTSC
